# Spectroscopic and Computational Characterization of 2-Aza-1,3-butadiene, a Molecule of Astrochemical Significance

**DOI:** 10.1021/acs.jpca.2c00831

**Published:** 2022-03-11

**Authors:** Ningjing Jiang, Mattia Melosso, Luca Bizzocchi, Silvia Alessandrini, Jean-Claude Guillemin, Luca Dore, Cristina Puzzarini

**Affiliations:** † Dipartimento di Chimica “Giacomo Ciamician”, Università di Bologna, Via F. Selmi 2, 40126 Bologna, Italy; ‡ Scuola Superiore Meridionale, Università di Napoli Federico II, Largo San Marcellino 10, 80138 Naples, Italy; § Scuola Normale Superiore, Piazza dei Cavalieri 7, 56126 Pisa, Italy; ∥ University of Rennes, Ecole Nationale Supérieure de Chimie de Rennes, CNRS, ISCR-UMR6226, F-35000 Rennes, France

## Abstract

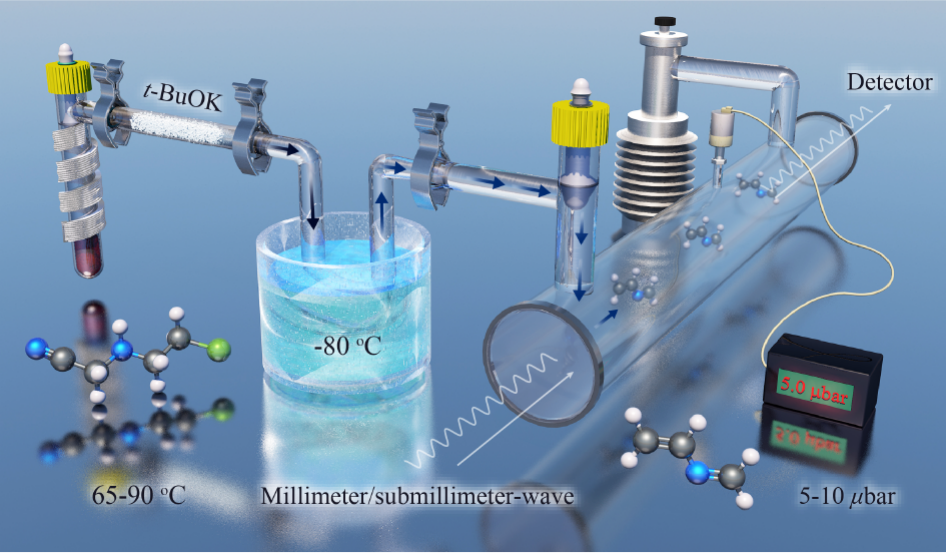

Being N-substituted unsaturated species, azabutadienes are molecules of potential relevance in astrochemistry, ranging from the interstellar medium to Titan’s atmosphere. 2-Azabutadiene and butadiene share a similar conjugated π system, thus allowing investigation of the effects of heteroatom substitution. More interestingly, 2-azabutadiene can be used to proxy the abundance of interstellar butadiene. To enable future astronomical searches, the rotational spectrum of 2-azabutadiene has been investigated up to 330 GHz. The experimental work has been supported and guided by accurate computational characterization of the molecular structure, energetics, and spectroscopic properties of the two possible forms, *trans* and *gauche*. The *trans* species, more stable by about 7 kJ/mol than *gauche*-2-azabutadiene, has been experimentally observed, and its rotational and centrifugal distortion constants have been obtained with remarkable accuracy, while theoretical estimates of the spectroscopic parameters are reported for *gauche*-2-azabutadiene.

## Introduction

1,3-Butadiene (CH_2_=CH—CH=CH_2_, hereafter denoted as butadiene) is a textbook species for understanding conjugation effects and, being the simplest conjugated diene, represents the prototypical reactant for Diels–Alder reactions. Molecules that are isoelectronic to butadiene and share a similar conjugated π system form an interesting family of species. Indeed, they allow the investigation of the heteroatom effects in the butadiene skeleton.^1^ Limiting ourselves to the second row of the periodic table, there are three singly substituted species isoelectronic to butadiene: acrolein (O=CH—CH=CH_2_, also known as propenal), 1-aza-1,3-butadiene (NH=CH—CH=CH_2_, hereafter 1-azabutadiene), and 2-aza-1,3-butadiene (CH_2_=N—CH=CH_2_, hereafter 2-azabutadiene). In this context, high-resolution molecular spectroscopy and computational chemistry are among the most powerful tools to elucidate the molecular structure, internal dynamics, and conformational behavior of these species, especially when the two approaches are combined synergistically.^2^

These butadiene-like species and butadiene itself are not easy to characterize experimentally. For example, due to the lack of a permanent electric dipole moment, the most stable *trans* form of butadiene does not possess any rotational spectrum. Hence, it is not possible to take advantage of the inherent accuracy of rotational spectroscopy to determine its molecular structure. In such a case, a rigorous geometrical determination could only be achieved by the analysis of ro-vibrational spectra for several isotopic species.^3−7^ As far as *gauche*-butadiene is concerned, its higher energy with respect to the *trans* form (∼12 kJ/mol) together with its small dipole moment value (0.09 D) have prevented its nonplanarity from being established unambiguously until recently.^8^

Whenever a heteroatom is introduced into the butadiene skeleton, the molecular symmetry is noticeably reduced, the resulting asymmetry then generating both an appreciable electric dipole moment and an increased number of active infrared modes. For these reasons, acrolein, 1-azabutadiene, and 2-azabutadiene represent more suitable spectroscopic targets. For example, owing to its large dipole moment values (about 3 and 2.5 D for the *trans* and *cis* forms, respectively^9^), acrolein has been well characterized from a spectroscopic point of view, as demonstrated by the vast literature on this subject.^10−16^ On the other hand, spectroscopic study of 1- and 2-azabutadiene has been quite limited to date^17−21^ as both species are unstable and require an efficient and selective production method.

Exhaustive characterization of the high-resolution rotational spectra for these butadiene-like species is also crucial for a different aspect: the study of the chemistry occurring in the interstellar medium (ISM). Despite the harsh conditions of molecular clouds, i.e., very low temperatures and number densities, unsaturated carbon chains are often abundant (see, for example, the case of TMC-1^22^ or Lupus-1A^23^), thus making butadiene and heterodienes good candidates for astronomical searches. However, because of its centrosymmetric nature, *trans*-butadiene cannot be detected in space through rotational emission and observation of the more energetic *gauche* form in cold molecular clouds is very unlikely. The abundance of centrosymmetric species, such as molecular nitrogen, cyanogen, or benzene, has been indirectly estimated through observation of their protonated,^24^ isomeric,^25^ or functionalized forms.^26^ Analogously, we suggest that the presence of interstellar butadiene can be proxied by the detection of its isoelectronic heterodienes. This strategy represents a promising route given the fact that acrolein has been recently discovered in the ISM^27^ and the detection of 1-azabutadiene is being reported tentatively.^28^ However, to the best of our knowledge, astronomical searches of 2-azabutadiene have never been reported. This is probably due to the fact that in addition to the limited spectral data reported in the literature, no entry for 2-azabutadiene is available in the widely used CDMS database^29,30^ or in the JPL catalog.^31^

In this respect, here, we report a refined analysis of the rotational spectrum of 2-azabutadiene based on new rotational transitions recorded between 225 and 330 GHz. The experimental work has been supported by high-level quantum-chemical calculations, which guided us through the spectral interpretation and provided a good reference set of spectroscopic parameters. This manuscript is organized as follows. In the next two sections, quantum-chemical computations and experimental details are discussed. Then, the results on 2-azabutadiene are presented in the fourth section, while a comparison with other dienes is discussed in the last section.

## Computational Details

The leading terms in rotational spectroscopy are the rotational constants, which can be computationally predicted by summing two contributions: the equilibrium rotational constants (*B*_e_), straightforwardly derived from the equilibrium structure, and the vibrational corrections (*ΔB*_vib_)
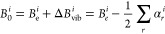
1In the equation above, *i* denotes the inertial axis (*i* = *a*, *b*, *c*, that is, for instance, *B*_0_^*a*^ = *A*), and the sum runs over all vibrational normal modes. Within vibrational perturbation theory to second order (VPT2), the vibrational corrections require computation of the vibration–rotation interaction constants (α_*r*_^*i*^).^32^ Their evaluation requires an anharmonic force field,^33^ which has been calculated at the MP2/cc-pVTZ^34^ level, within the frozen-core (fc) approximation. The MP2 acronym stands for Møller–Plesset perturbation theory to the second order.^35^

Despite being important for quantitative predictions,^36,37^ the vibrational corrections provide a small contribution to the *B*_0_ constants, indeed accounting only for ∼1–3% of the total value. This implies that the equilibrium structure should be evaluated with high accuracy. For this purpose, the so-called CBS+CV composite scheme has been employed,^38^ which is based on the CCSD(T) method (coupled-cluster (CC) singles and doubles with perturbative treatment of triples^39^) and requires minimization of the following energy gradient
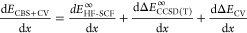
2where d*E*_HF–SCF_^∞^/d*x* and d*ΔE*_CCSD(T)_^∞^/d*x* are gradients for the extrapolation to the complete basis set (CBS) limit of the HF-SCF energy (exponential extrapolation formula by Feller^40^) and of the CCSD(T) electron correlation contribution (*n*^–3^ extrapolation expression^41^), respectively. Since the extrapolation to the CBS limit is performed within the frozen core approximation, core–valence (CV) correlation effects are incorporated by adding the corresponding correction, d*ΔE*_CV_/d*x*. This involves the difference of all-electron (ae) and frozen-core CCSD(T) calculations using the same basis set. In the CBS+CV scheme, for the extrapolation to the CBS limit, we used the cc-pV*n*Z basis sets,^34^ with *n* = T – 5 for HF-SCF and *n* = T, Q for CCSD(T). For the CV correction, we resorted to the cc-pCVTZ basis set.^42^

The semirigidity of the molecules also requires one to account for the effect of centrifugal distortion. Different orders of centrifugal distortion terms are possible according to the power of the angular momentum operator considered in the effective rotational Hamiltonian. However, from a computational point of view, only the quartic and sextic centrifugal-distortion constants can be obtained: the former as a byproduct of harmonic force-field computations, and the latter from the anharmonic force field. Therefore, the sextic terms have been computed at the MP2/cc-pVTZ level, while the quartic centrifugal-distortion constants have been purposely derived from a fc-CCSD(T)/cc-pVTZ harmonic force field. This latter calculation also allowed for determining the electric dipole moment (μ) components as well as the electric field gradients at the nitrogen nucleus, which straightforwardly provide the nitrogen quadrupole coupling constants.^33^

All quantum-chemical computations have been carried out with the CFOUR package.^43,44^

## Experimental Section

The sample of 2-azabutadiene was synthesized in a two-step procedure, and its rotational spectrum was recorded with a frequency-modulation spectrometer.

The synthetic reaction reported in the literature for obtaining 2-azabutadiene^45,46^ has been slightly revised, and the experimental conditions were optimized with the aim of maximizing the signal-to-noise ratio (S/N) of the rotational spectrum and minimizing the number of possible interfering lines. The production of 2-azabutadiene proceeds in two steps, detailed in the following: synthesis of the precursor (step I) and its transformation into the title molecule (step II).

### Step I: Synthesis of the Precursor

The precursor is 2-[(2-chloroethyl)amino]acetonitrile, which has been synthesized in a Strecker reaction starting from 2-chloroethylamine hydrochloride (0.1 mol, 11.6 g) and sodium cyanide (0.1 mol, 5 g) in methanol (30 mL) and water (20 mL). The reaction mixture was cooled to 0 °C, and formaldehyde (37% in water, 0.1 mol) was added slowly. The solution was allowed to warm up to room temperature and stirred for 1 h. Organic compounds were then extracted with dichloromethane (3 × 50 mL). The organic phase was dried over magnesium sulfate, and the solvents were removed in vacuo. The crude product obtained in a 90% yield (10.6 g) was sufficiently pure to be used directly in the next step.^45,46^ Distillation under vacuum (0.1 mbar) gave the pure product in a 43% yield (5.1 g).

2-Chloroethylamine hydrochloride, formaldehyde in water, and sodium cyanide were purchased from Sigma-Aldrich and used without further purification.

### Step II: Synthesis of 2-Azabutadiene

2-Azabutadiene was synthesized by vaporizing 2-[(2-chloroethyl)amino]acetonitrile (2 g) on potassium *tert*-butylate (*t*-BuOK, 30 g) introduced in a horizontal tube and filling its lower half. In this reaction, *tert*-butanol was also produced and selectively trapped in a U-tube immersed in a cooling bath, while a gaseous flow of almost pure 2-azabutadiene was directly introduced into the spectrometer cell.

**Figure 1:**
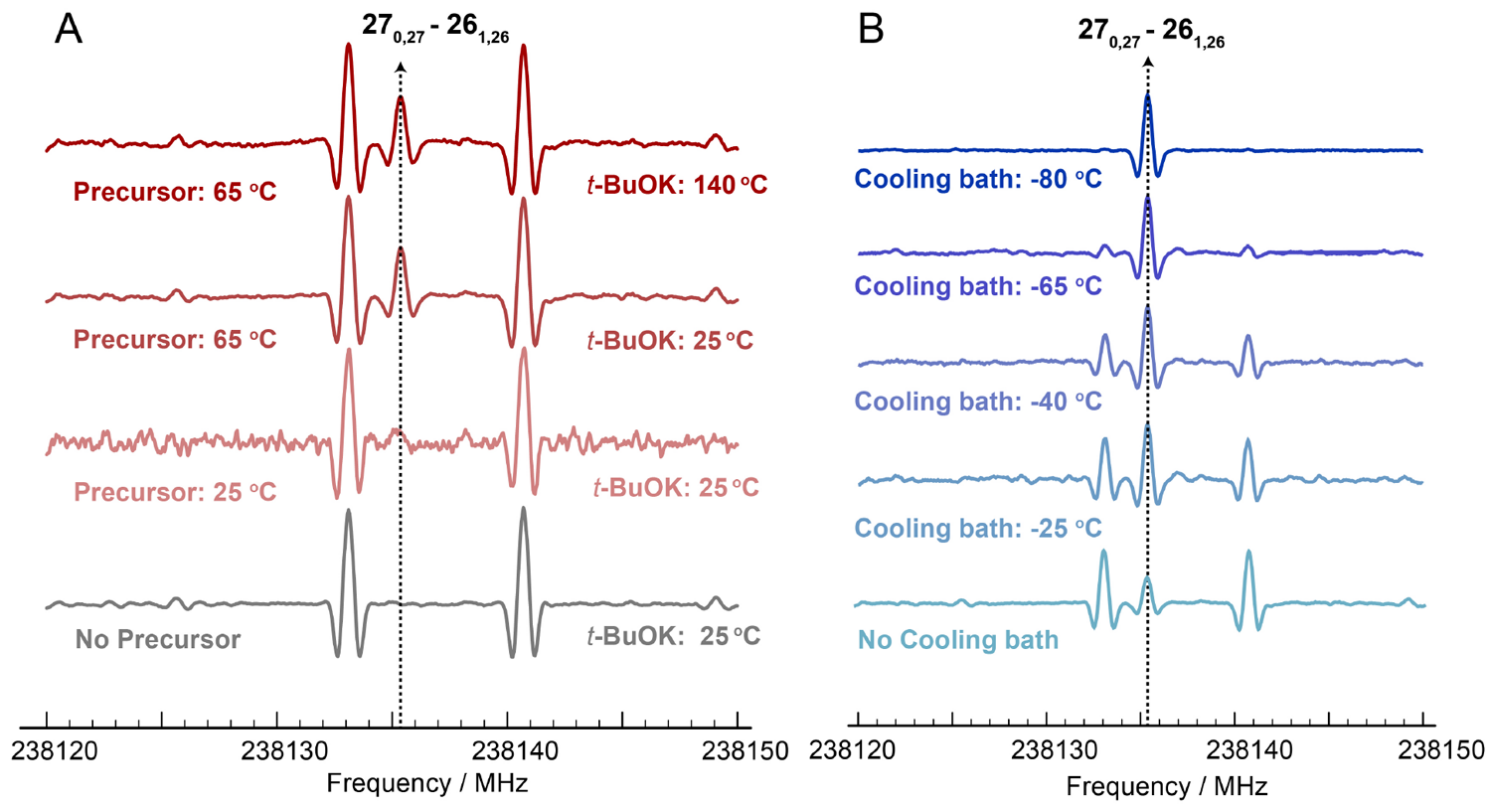
Portion of the rotational spectra around the *J*_*Ka*,*Kc*_ = 27_0,27_ ← 26_1, 26_ transition of *trans*-2-azabutadiene recorded while optimizing the experimental conditions. Two lines adjacent to the one of interest belong to *t*-BuOH, formed during the reaction. (A) Several spectra acquired during optimization of the reactant temperatures, whereas (B) spectra recorded keeping 2-[(2-chloroethyl)amino]acetonitrile at 65 °C and *t*-BuOK at room temperature while varying the temperature of the cooling bath.

The best conditions were optimized by monitoring the spectral S/N for the *J*_*Ka*, *Kc*_ = 27_0,27_ ← 26_1,26_ transition of *trans*-2-azabutadiene, predicted on the basis of literature data.^20^Figure 1 shows several spectra recorded upon changing the temperature of (i) the precursor species, (ii) *t*-BuOK, and (iii) the cooling bath. Ultimately, the maximum yield of 2-azabutadiene (and consequently the best S/N in the spectrum) was achieved by maintaining the precursor compound between 65 and 90 °C, the potassium *tert*-butylate sample at room temperature, and the cooling bath at −80 °C. The cooling bath was obtained by mixing acetone with liquid nitrogen and allowed for complete removal of the interfering lines belonging to the *t*-BuOH formed during the reaction.

### Frequency-Modulation Millimeter Spectrometer

The rotational spectrum of 2-azabutadiene has been recorded between 225 and 330 GHz by means of a frequency-modulation millimeter-wave spectrometer in Bologna; a detailed description of the instrument is given in previous works.^47,48^ Briefly, the primary radiation source—whose frequency and phase stability are ensured by a phase-lock loop (PLL)—is a Gunn diode emitting in the W band (75–110 GHz). The Gunn diode is coupled to a passive multiplier (tripler, WR3.4x3 Virginia Diodes Inc.) to produce higher frequencies. The modulated radiation source is fed into a 3 m glass absorption cell where the vapors of 2-azabutadiene are injected at a pressure between 5 and 10 μbar. The output signal is detected by a Schottky barrier diode and demodulated by a lock-in amplifier set at twice the modulation frequency (2*f*). The uncertainties of the present experiment are around 30 kHz.

## Results

**Figure 2:**
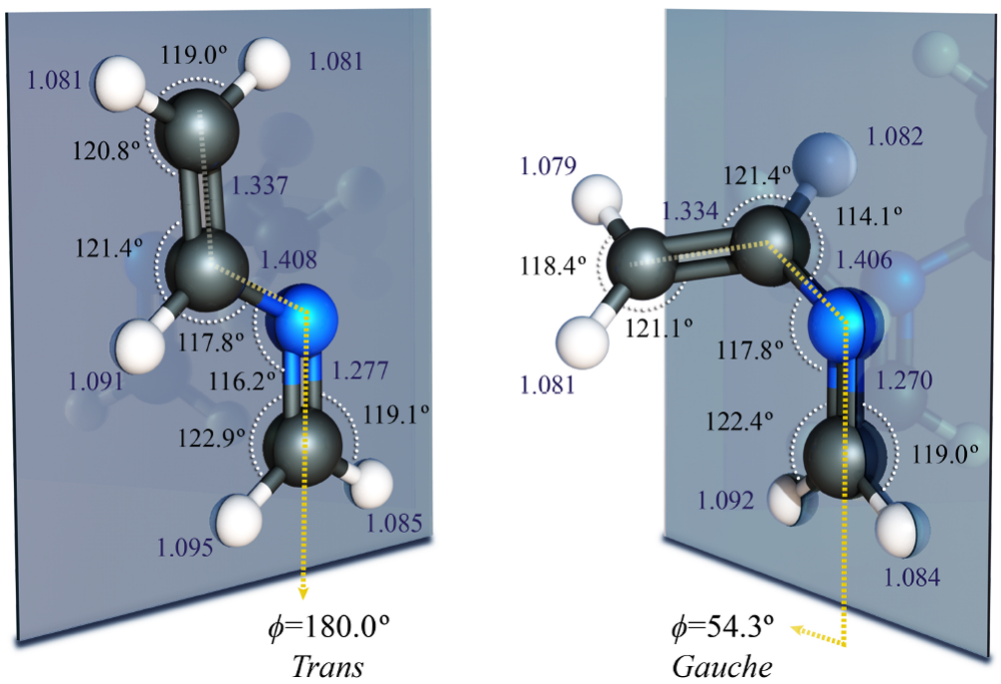
Molecular structures of *trans*- and *gauche*-2-azabutadiene together with the optimized geometrical parameters (CBS+CV level, see text; bond lengths in Angstroms; angles in degrees).

2-Azabutadiene exists in two conformations, depending on the C=N—C=C dihedral angle (ϕ): the most stable planar *trans* form (ϕ = 180°) and the more energetic *gauche* isomer (ϕ = 54°). The optimized equilibrium structures are shown in Figure 2 together with the corresponding CBS+CV geometrical parameters (Cartesian coordinates are available in the Supporting Information). Except for the dihedral angle, most of the bond lengths and angles are similar in the two forms, i.e., they agree within few milliAngstroms for the former and ∼1° for the latter. The largest differences are observed for the H–C=N angle (3.7°) and the two involved bonds (7 mÅ for C=N and 9 mÅ for C–H). Understanding the reasons at the basis of these differences is beyond the scope of our paper; however, they can be simply rationalized by performing natural bond orbital (NBO) analysis. The results of such analysis are collected in the SI and can be used to understand how the conjugation effects stabilize the *trans* species more than the *gauche* form.

At the CBS+CV level, the equilibrium energy difference (*ΔE*[*gauche – trans*]) between the two conformers is 6.5 kJ/mol. By incorporating the harmonic zero-point energy (ZPE) correction, computed at the fc-CCSD(T)/cc-pVTZ level of theory, *ΔE*[*gauche – trans*] increases to 7.2 kJ/mol. On the basis of this energy difference, the population of *gauche*-2-azabutadiene is about 7% of that of the *trans* form at room temperature. Since the computed (μ_*b*_^(gauche)^/μ_*b*_^(trans)^)^2^ ratio is ∼0.7 (see Table 1), rotational transitions belonging to *gauche*-2-azabutadiene are expected to be roughly 20 times weaker than those of the *trans* species. Even though the attained S/N of the spectrum is about 150 for *trans*-2-azabutadiene, this is expected to be less than 10 for the *gauche* form. In addition, the *trans* species possesses eight vibrational states lying below the energy of *gauche*-2-azabutadiene. Therefore, in conclusion, identification of the weak spectrum of the *gauche* form is prevented by the huge number of interfering lines, and hereafter, we will focus on the only conformer securely characterized in this work, i.e., the *trans* species. Nonetheless, the theoretical spectroscopic constants provided in this manuscript (see Table 1) represent a solid base for attempting the identification of *gauche*-2-azabutadiene in different experiments (e.g., FTMW spectroscopy of a jet-cooled sample).

**Table 1:** Ground-State Spectroscopic Parameters of *trans*- and *gauche*-2-Azabutadiene (*S* Reduction, *I*^*r*^ Representation)

		trans			gauche	
parameters	units	exp.^a^	theo.^b^	ref ^20^^c^	theo.^b^	scaled^d^
*A*	MHz	47 186.0004(7)	47231.509	47 186.012(7)	24 021.128	23 997.983
*B*	MHz	4886.5219(1)	4887.740	4886.5223(8)	6071.991	6070.478
*C*	MHz	4430.0772(1)	4430.824	4430.0778(8)	5120.829	5119.966
*D*_*J*_	kHz	0.97917(8)	0.960416	0.980(3)	5.42141	5.52727
*D*_*JK*_	kHz	–8.0641(7)	–7.38104	–7.99(9)	–40.8174	–44.5947
*D*_*K*_	kHz	341.95(2)	309.12	341.(1)	207.669	229.722
*d*_1_	kHz	–0.117415(5)	–0.120169	–0.1173(1)	–1.82008	–1.77837
*d*_2_	kHz	–0.006949(1)	–0.006235	–0.00695(2)	–0.19994	–0.22283
*H*_*J*_	mHz	0.29(1)	0.217		–12.7	–16.97
*H*_*JK*_	mHz	–8.0(2)	–3.856		308.2	639.4
*H*_*KJ*_	Hz	–0.267(2)	–0.411		–3.736	–2.431
*H*_*K*_	Hz	1.3(1)	5.75		14.019	3.167
*h*_1_	mHz	0.0691(9)	0.0786		–7.35	–6.462
*h*_2_	mHz	0.0104	0.0104		–1.12	–1.12
*h*_3_	mHz	0.0016	0.0016		–0.0691	–0.0691
1.5 × χ_*aa*_	MHz	1.411	1.411	0.8(15)	1.275	1.275
(χ_*bb*_ – χ_*cc*_)/4	MHz	–2.142(7)	–2.13	–2.1(12)	–0.839	–0.839
|μ_*a*_|	D	0.44(3)^e^	0.47		0.029	0.027
|μ_*b*_|	D	1.90(7)^e^	1.46		1.206	1.569
|μ_*c*_|	D				0.806	0.806
no. of data		675		82		
*J*_max_, *K*_a max_		76, 20		31, 4		
rms error	kHz	32.9		31.8		
σ		1.09		1.06		

a Values in parentheses are one standard deviation and refer to the last digits. Parameters without uncertainties are held fixed at the corresponding computed value.

b CBS+CV equilibrium rotational constants augmented by vibrational corrections at the fc-MP2/cc-pVTZ level. Quartic centrifugal distortion and nuclear quadrupole coupling constants computed at the fc-CCSD(T)/cc-pVTZ level. Sextic centrifugal distortion terms at the fc-MP2/cc-pVTZ level. Equilibrium dipole moment components calculated at the fc-CCSD(T)/cc-pVTZ level and augmented by vibrational corrections at the fc-MP2/cc-pVTZ level.

c Converted from *A* to *S* reduction.

d Obtained using eq 3. See text for details.

e Determined by Stark effect measurements in ref (20).

Moving to the fully characterized *trans*-2-azabutadiene species, this is an asymmetric rotor with a strong nearly prolate nature (κ = −0.979). In its principal inertia system, the molecule lies on the *ab* plane with the *c* axis perpendicular to it. The computed dipole moment components are μ_*a*_ = 0.47 D and μ_*b*_ = 1.46 D (see Table 1), with μ_*c*_ being null for symmetry reasons (the point group symmetry is *C*_*s*_).

**Figure 3:**
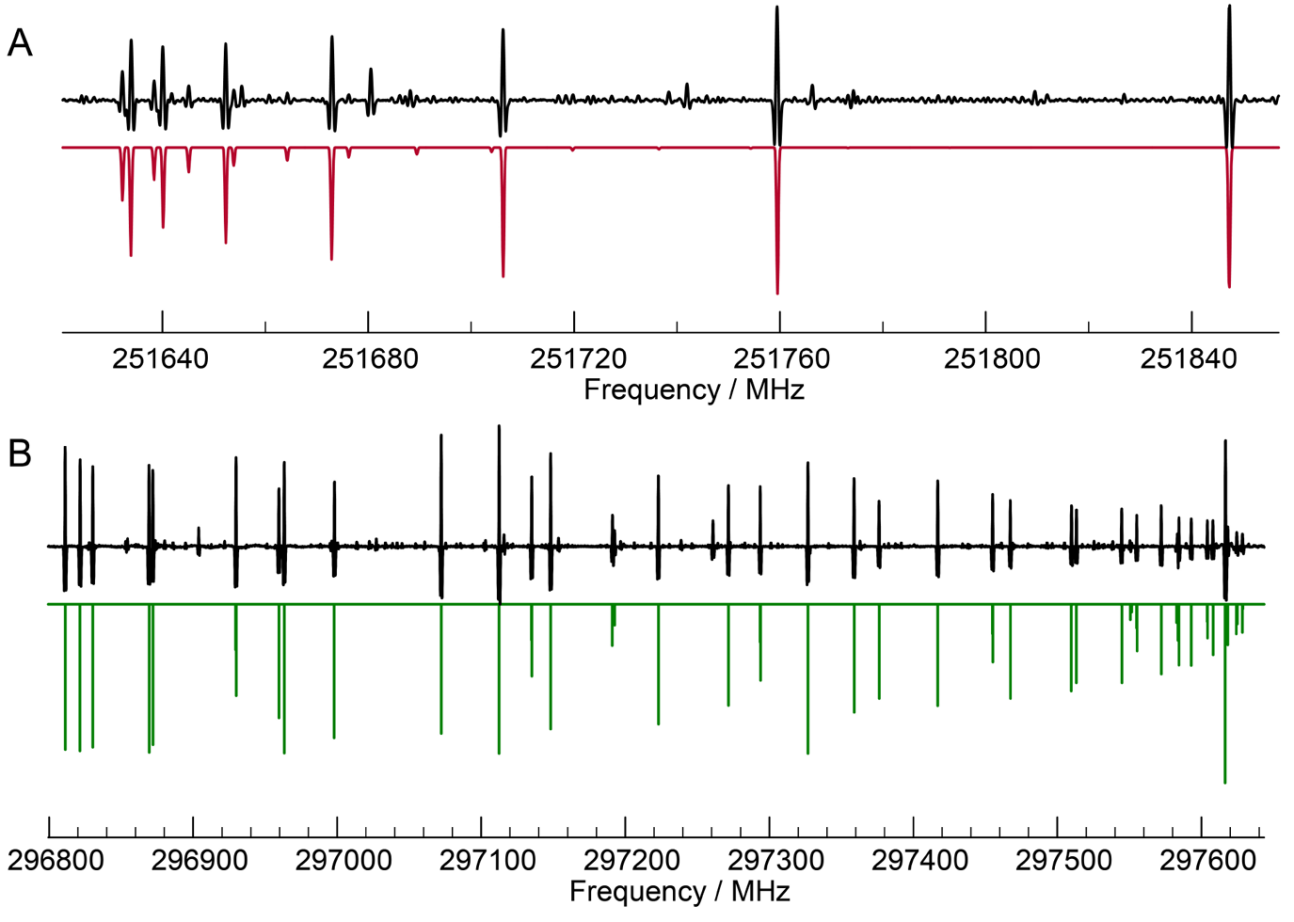
Portion of the rotational spectrum of 2-azabutadiene (black). Red trace depicts *a*-type transitions (A), while green trace represents *b*-type transitions (B).

Exploiting the best experimental conditions, almost 600 new rotational transitions, both of *a* and of *b* type, were recorded between 225 and 330 GHz (examples are provided in Figure 3). They probe energy levels with *J* up to 76 and *K*_*a*_ up to 20, such a difference in the upper limits of *J* and *K*_*a*_ being ascribable to the relatively large *A* rotational constant (∼47 GHz) with respect to the much smaller *B* and *C* values (∼4.4 GHz). Indeed, the population of the rotational energy levels decreases quickly by increasing *K*_*a*_ and slowly by increasing *J*. Our newly observed rotational transitions were combined with previous low-frequency data^20^ and analyzed in a weighted least-squares procedure using the SPFIT program.^49^ The optimized energy level positions were calculated employing a Watson *S*-reduced Hamiltonian with centrifugal distortion terms up to the sixth power of the angular momentum operator.^50^ Despite the relatively high *J* values targeted in this work, the hyperfine structure arising from the quadrupolar ^14^N nucleus has been partially resolved for some low *K*_*a*_, *b*-type transitions. Therefore, we were able to determine the (χ_*bb*_ – χ_*cc*_)/4 parameter, whereas the value of 1.5 × χ_*aa*_ has been kept fixed to its computed value. The derived spectroscopic parameters are collected in Table 1, where theoretical estimates are also provided.

From inspection of Table 1, we note that the root-mean-square error of the residuals (32.9 kHz) is in line with the estimated accuracy of both our transition frequencies and those reported in the literature.^20^ The semirigid model is appropriate for the description of the rotational energy levels, as demonstrated by the standard deviation of the fit (σ), which is 1.09. The rotational constants *A*, *B*, and *C* have been determined with remarkably good accuracy, the associated errors being smaller than 1 kHz. Notably, all quartic centrifugal distortion constants have been obtained with an uncertainty smaller than 0.01%, and most of the sextic terms could be derived with the exception of two off-diagonal constants (*h*_2_ and *h*_3_) that have been kept fixed at the corresponding computed values. The agreement between the experimental and the theoretical parameters is very good. Minor discrepancies are only found for the sextic constants and the value of the *b* component of the dipole moment, experimentally determined by means of Stark effect measurements.^20^ The latter discrepancy is unexpected based on the literature available for dipole moment computations.^51^ Indeed, even though the use of augmented basis sets is recommended to ensure a flexible description of the outer valence region, the accuracy of the fc-CCSD(T)/cc-pVTZ values is expected to be on the order of the experimental uncertainty.^51^ In this respect, the limitations of the experimental determination deserve to be mentioned; in fact, for both μ_*a*_ and μ_*b*_, this relied on the observation of only two transitions.

The improvement in the accuracy of our spectroscopic parameters over those derived by Sugie et al.^20^ is evident (see Table 1); the uncertainty on the rotational and centrifugal distortion constants has been reduced by 1 and 2 orders of magnitude, respectively. Owing to the higher *J* and *K*_*a*_ values sampled in our measurements and to the enlarged data set available, centrifugal analysis of *trans*-2-azabutadiene has been expanded and now includes distortion terms up to the sextic constants.

Concerning the *gauche* form of 2-azabutadiene, our ab initio estimate of the spectroscopic parameters should provide reliable spectral predictions with an overall uncertainty on transition frequencies of about 0.1–0.2%. This deviation can be further reduced by applying an empirical, well-tested, scaling procedure based on the ratio between the computed and the experimental constants for the *trans* form
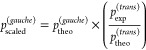
3where *p*^(X)^ represents the generic parameters of the X isomer. The scaled parameters are collected in the last column of Table 1, and their accuracy is expected to be increased by 1 order of magnitude with respect to the computed values.

## Discussion and Conclusions

Given the similar heavy-atom skeleton and the conjugated π system shared by 2-azabutadiene, butadiene, acrolein, and 1-azabutadiene, it is quite interesting to qualitatively compare the values of the rotational and centrifugal distortion constants available for each species. This comparison is performed separately for the *trans* and *cis/gauche* species, the results being reported in Tables 2 and 3, respectively. As far as the *trans* species are concerned, the similarity is evident. The order of magnitude and the sign of each constant listed in Table 2 are the same for all of the species considered. This indicates a similar mass distribution (i.e., molecular geometry) and stiffness of the chemical bonds due to the analogy between the conjugated systems.

**Table 2:** Rotational and Quartic Centrifugal Distortion Constants of the *Trans* Species^a^

		butadiene^b^	1-azabutadiene^c^		acrolein^d^	2-azabutadiene
constant	units		anti	syn		
*A*	MHz	41 682.6292(11)	45 773.6451(9)	43 755.656(2)	47 353.7034(20)	47 186.0004(7)
*B*	MHz	4433.50067(18)	4560.93131(7)	4564.54014(7)	4659.48810(18)	4886.5219(1)
*C*	MHz	4008.04876(19)	4148.24852(6)	4134.46273(6)	4242.70127(16)	4430.0772(1)
*D*_*J*_	kHz	0.861735(27)	0.90964(3)	0.95037(4)	1.028148(56)	0.97917(8)
*D*_*JK*_	kHz	–7.15724(48)	–7.531(1)	–7.589(1)	–8.70159(74)	–8.0641(7)
*D*_*K*_	kHz	218.6356(33)	296.80(3)	257.29(7)	360.142(30)	341.95(2)
*d*_1_	kHz	–0.107889(16)	–0.10715(1)	–0.114863(5)	–0.120228(21)	–0.117415(5)
*d*_2_	kHz	–0.0056450(72)	–0.005863(2)	–0.006237(3)	–0.0069896(77)	–0.006949(1)

a Values in parentheses are one standard deviation and refer to the last digits.

b From ref (3).

c From ref (28).

d From ref (16). Converted from *A* to *S* reduction.

**Table 3:** Rotational and Quartic Centrifugal Distortion Constants of the *Cis* and *Gauche* Species^a^

		butadiene^b^		acrolein^c^	2-azabutadiene
constant	units	0^+^ state	0^–^ state		
*A*	MHz	21 223.0467(18)	21 232.3005(80)	22 831.6513(51)	24 021.128
*B*	MHz	5671.39702(56)	5667.6405(10)	6241.02408(38)	6071.991
*C*	MHz	4577.35202(47)	4581.14000(70)	4902.23005(24)	5120.829
*D*_*J*_	kHz	4.630(34)	4.630(34)	4.893610(178)	5.42141
*D*_*JK*_	kHz	–6.99(24)	97.67(40)	–27.8640(13)	–40.8174
*D*_*K*_	kHz	114.65(32)	1039.6(76)	106.63(13)	207.669
*d*_1_	kHz	–1.2045(112)	–1.932(28)	–1.480975(128)	–1.82008
*d*_2_	kHz	0.635(41)	–3.57(17)	–0.109707(78)	–0.199939

a Values in parentheses are one standard deviation and refer to the last digits.

b From ref (8).

c From ref (16). Converted from *A* to *S* reduction.

The case of the *gauche/cis* species is slightly different. Here, the similarity is less pronounced due to two facts. First, the higher energy conformer of acrolein displays a planar cis geometry, while both butadiene and 2-azabutadiene possess nonplanar gauche forms. Second, *gauche*-butadiene exhibits tunneling between two equivalent minima, whereas no large amplitude motion occurs in the heterodienes. That being said, it is likely that the differences observed for the two inversion states of butadiene are due to an unsatisfactory treatment of the Coriolis interaction occurring between the 0^+^ and the 0^–^ states. In this framework, the centrifugal distortion constants of acrolein and 2-azabutadiene might be used as a reference to guide the analysis toward a better modeling.

Centrifugal analysis of 2-azabutadiene has another important outcome. The rotational spectrum of the most stable *trans* form now can be predicted with high accuracy in a wide range of frequencies. This represents a key prerequisite for enabling astronomical searches of this species in dense spectral line surveys of molecular-rich objects, where an unambiguous spectroscopic identification typically requires a match within a few tens of kilohertz.

One isomer of 2-azabutadiene, namely, ethyl cyanide, has been observed in high-mass star-forming regions with remarkable high abundance,^52−55^ while another isomer, i.e., 1-azabutadiene, is reported as tentatively detected in G+0.693.^28^ These discoveries tell us that molecules showing the same degree of complexity as 2-azabutadiene can be formed efficiently in interstellar conditions, thus making the title heterodiene a good candidate for future detection. Besides giving information about the kinetics and thermodynamics of the C_3_H_5_N isomeric family, 2-azabutadiene can be used—together with the other heterodienes—to proxy the abundance of interstellar butadiene or even ethene. While we do not have yet any evidence that this suggestion might work, indirect support to this idea is provided by the relative abundance of CCH over CN cyclic-hydrocarbon derivatives in TMC-1, which are highly correlated.^56^ In work by Cernicharo et al.,^56^ it was also suggested that their relative abundance probably reflected the abundance of the CCH and CN radicals. In this respect, we note that 2-azabutadiene can be seen as substituted ethene, where the H_2_CN radical is linked to the latter through the N atom.

Finally, Titan’s chemistry deserves a note. Organic nitriles and, more generally, N-substituted hydrocarbons are largely present in the nitrogen-rich atmosphere Titan. In this view, spectroscopic characterization of 2-azabutadiene might be useful to gain further information on the chemistry occurring in what is considered to resemble the primitive Earth.

## Supporting Information Available

Cartesian coordinates of the CBS+CV molecular structure for both *trans* and *gauche* forms; results of the NBO analysis; harmonic and anharmonic vibrational frequencies, IR intensities, and vibration–rotation α constants; ^1^H and ^13^C NMR spectra of 2-[(2-chloroethyl)amino]acetonitrile and 2-azabutadiene; list of assigned rotational transitions together with the corresponding residuals from the final fit (PDF)
