# Supplementary material for: Spectroscopic and Computational Characterization of 2-Aza-1,3-butadiene, a Molecule of Astrochemical Significance
Source: J Phys Chem A. 2022 Mar 11;126(11):1881–8. doi: 10.1021/acs.jpca.2c00831 (PMC8958585; doi:10.1021/acs.jpca.2c00831)
Supplement: Supplementary file 1 — jp2c00831_si_001.pdf [file jp2c00831_si_001.pdf]

Supporting Information: Spectroscopic and Computational Characterization of 2-aza-1,3-butadiene, a Molecule of Astrochemical Significance

Ningjing Jiang,† Mattia Melosso,*,†,‡ Luca Bizzocchi,† Silvia Alessandrini,¶ Jean-Claude Guillemin,§ Luca Dore,† and Cristina Puzzarini*,†

†*Dipartimento di Chimica "Giacomo Ciamician", Università di Bologna, Via F. Selmi 2, 40126 Bologna, Italy*

‡*Scuola Superiore Meridionale, Università di Napoli Federico II, Largo San Marcellino 10, 80138 Naples, Italy*

¶*Scuola Normale Superiore, Piazza dei Cavalieri 7, 56126 Pisa, Italy*

§*Univ Rennes, Ecole Nationale Supérieure de Chimie de Rennes, CNRS, ISCR–UMR6226, F-35000 Rennes, France*

E-mail: mattia.melosso2@unibo.it; cristina.puzzarini@unibo.it

Quantum-Chemical Calculations

Molecular Structures

The tables here below collect the Cartesian coordinates of *trans*- and *gauche*-2-azabutadiene resulting from the geometry optimization exploiting the so-called CBS+CV composite scheme (which is detailed in the main text).

Table S1: Cartesian coordinates (in Å units) of the equilibrium CBS+CV molecular structure of *trans*-2-azabutadiene.

Atom	Numbering	X	Y	Z
C	1	-1.71011	0.12067	0.00000
N	2	-0.57211	-0.45098	0.00000
H	3	-1.82638	1.20772	0.00000
H	4	-2.60549	-0.48873	0.00000
C	5	0.55033	0.39155	0.00000
H	6	0.37760	1.46737	0.00000
C	7	1.78346	-0.11482	0.00000
H	8	2.64664	0.53285	0.00000
H	9	1.93062	-1.18477	0.00000

Table S2: Cartesian coordinates (in Å units) of the equilibrium CBS+CV molecular structure of *gauche*-2-azabutadiene.

Atom	Numbering	X	Y	Z
C	1	-1.42122	-0.47543	0.23080
N	2	-0.70711	0.51512	-0.42880
C	3	0.64254	0.51956	0.31276
H	4	-1.03860	-1.27975	1.42511
H	5	-2.45071	-0.52436	-0.40333
H	6	0.96462	1.44359	1.18595
C	7	1.50251	-0.46915	-0.16213
H	8	2.54084	-0.37491	0.36299
H	9	1.19027	-1.36122	-1.15446

NBO analysis

The natural bond orbital (NBO) analysis [1] has been performed at the B3LYP-D3(BJ)/jun-cc-pVTZ level [2-4] using the fc-CCSD(T)/cc-pVTZ geometry. The Gaussian 16 [5] has been employed.

Table S3: Second-order perturbation theory analysis of Fock matrix in NBO basis for 2-azabutadiene (threshold for printing: 4 kcal/mol).

Donor (L) NBO		Acceptor (NL) NBO		E(2) (kcal/mol)
		trans		
LP(1)	N2	BD*(1)	C1-H3	9.93
LP(1)	N2	BD*(1)	C1-H4	5.36
LP(1)	N2	BD*(1)	C5-H6	7.71
LP(1)	N2	RY (1)	C1	4.13
BD(2)	C1-N2	BD*(2)	C5-C7	16.67
BD(1)	C1-H4	BD*(1)	N2-C5	5.27
BD(2)	C5-C7	BD*(2)	C1-N2	14.17
BD(1)	C7-H8	BD*(1)	N2-C5	5.64
BD(1)	C7-H9	BD*(1)	C5-H6	4.93
		gauche		
LP(1)	N2	BD*(1)	C1-H4	10.27
LP(1)	N2	BD*(1)	C1-H5	5.47
LP(1)	N2	BD*(1)	C3-C7	7.70
BD(2)	C1-N2	BD*(2)	C3-C7	9.37
BD(1)	C1-H5	BD*(1)	N2-C3	5.55
BD(1)	C3-H6	BD*(1)	C7-H9	4.06
BD(2)	C3-C7	BD*(2)	C1-N2	7.73
BD(1)	C7-H8	BD*(1)	N2-C3	6.19
BD(1)	C7-H9	BD*(1)	C3-H6	4.66

References

1. E.D. Glendening, C. R. Landis, F. Weinhold, Wiley Interdiscip. Rev.: Comput. Mol. Sci. 2012, 2, 1-42.

2. a) A. D. Becke, J. Chem. Phys. 1993, 98, 5648-5652; b) P. J. Stephens, F. J. Devlin, C. F. Chabalowski, M. J. Frisch, J. Phys. Chem. 1994, 98, 11623-11627.

3. a) S. Grimme, J. Antony, S. Ehrlich, H. Krieg, J. Chem. Phys. 2010, 132, 154104; b) S. Grimme, S. Ehrlich, L. Goerigk, J. Comput. Chem. 2011, 32, 1456-1465.

4. a) E. Papajak, D. G. Truhlar, J. Chem. Theory Comput. 2011, 7, 10-18; b) E. Papajak, J. Zheng, X. Xu, H. R. Leverentz, D. G. Truhlar, J. Chem. Theory Comput. 2011, 7, 3027-3034.

5. Gaussian16, RevisionC.01, M. J. Frisch, G.W. Trucks, H. B. Schlegel, G. E. Scuseria, M. A. Robb, J. R. Cheeseman, G. Scalmani, V. Barone, G. A. Petersson, H. Nakatsuji et al., Gaussian, Inc., Wallingford CT, 2016.

Fundamental Frequencies and Vibration-rotation Interaction Constants

Harmonic and anharmonic force-field calculations have been carried out: the former at the fc-CCSD(T)/cc-pVTZ level and the latter at the fc-MP2/cc-pVTZ level. The reader is referred to the main text for all details. For *trans*- and *gauche*-2-azabutadiene, the tables here below collect the fc-CCSD(T)/cc-pVTZ fundamental harmonic frequencies, which have been corrected for anharmonic effects at the fc-MP2/cc-pVTZ level. Anharmonic force-field calculations also provide the vibration-rotation interaction constants (α), which are reported in the last three columns.

Table S4: Fundamental harmonic and anharmonic frequencies, harmonic IR intensities, and vibration-rotation α constants of *trans*-2-azabutadiene.

Mode	Symmetry	Harmonic[a] (cm^{-1})	Anharmonic[b] (cm^{-1})	Intensity[a] (km/mol)	α^{A}[b] (MHz)	α^{B}[b] (MHz)	α^{C}[b] (MHz)
ν_1	A'	3260.708	3122.046	2.126	-86.142	-3.431	-3.212
ν_2	A'	3179.595	3043.330	14.570	-28.895	-4.256	-3.419
ν_3	A'	3164.736	3048.293	1.224	-112.767	-2.606	-3.149
ν_4	A'	3093.314	2961.648	29.735	-68.519	-4.405	-3.981
ν_5	A'	3028.656	2885.550	41.738	-90.738	-2.244	-2.497
ν_6	A'	1677.347	1632.243	14.089	-49.060	-12.362	-11.946
ν_7	A'	1659.517	1615.536	7.502	-82.376	-8.141	-9.249
ν_8	A'	1487.676	1454.777	5.649	68.914	-0.964	-3.530
ν_9	A'	1427.145	1374.583	4.162	143.169	2.367	-2.473
ν_{10}	A'	1320.392	1294.994	3.730	43.661	2.678	-1.004
ν_{11}	A'	1256.284	1227.969	22.613	348.674	-3.662	-11.371
ν_{12}	A'	1116.303	1096.228	0.647	524.620	2.642	-7.833
ν_{13}	A'	913.614	897.367	3.572	-646.033	-15.360	-9.121
ν_{14}	A'	559.312	552.353	2.342	29.645	-6.423	-5.954
ν_{15}	A'	333.840	333.523	1.787	1229.766	0.379	-4.159
ν_{16}	A''	1037.650	1016.705	23.108	-592.352	-5.690	1.028
ν_{17}	A''	990.175	966.397	15.191	-252.181	-1.914	1.311
ν_{18}	A''	924.745	907.428	36.553	927.470	-2.579	0.862
ν_{19}	A''	810.894	793.761	0.005	-702.662	-3.224	1.106
ν_{20}	A''	605.003	587.189	1.919	-37.892	-6.734	-1.151
ν_{21}	A''	147.388	141.566	7.122	-1437.656	4.099	16.012

[a] At the fc-CCSD(T)/cc-pVTZ level.
[b] At the fc-MP2/cc-pVTZ level.

Table S5: Fundamental harmonic and anharmonic frequencies, harmonic IR intensities, and vibration-rotation α constants of *gauche*-2-azabutadiene.

Mode	Harmonic[a] (cm^{-1})	Anharmonic[b] (cm^{-1})	Intensity[a] (km/mol)	α^{A} [b] (MHz)	α^{B} [b] (MHz)	α^{C} [b] (MHz)
ν_1	3251.440	3112.714	8.159	-34.615	-3.321	-3.946
ν_2	3181.832	3045.840	6.518	5.277	-13.904	-7.069
ν_3	3173.785	3038.410	21.446	3.343	-8.405	-5.163
ν_4	3154.802	3045.835	2.464	-28.773	-3.794	-3.654
ν_5	3042.319	2901.837	30.438	-26.680	-1.554	-3.128
ν_6	1679.059	1633.763	19.183	-175.205	9.173	0.827
ν_7	1659.370	1615.844	28.942	-84.466	-4.391	-13.768
ν_8	1495.770	1458.825	13.315	19.608	3.103	-0.674
ν_9	1434.862	1398.762	1.457	44.849	2.591	-1.231
ν_{10}	1314.395	1288.622	1.708	-18.381	7.799	2.697
ν_{11}	1220.577	1197.752	12.832	71.225	1.865	-4.758
ν_{12}	1123.135	1094.015	12.266	53.147	-1.884	-7.635
ν_{13}	1057.543	1035.675	20.321	-127.331	-3.871	-1.219
ν_{14}	991.360	968.521	24.439	-49.617	-4.210	2.518
ν_{15}	917.678	897.490	10.196	89.410	-20.966	-15.205
ν_{16}	885.565	868.530	46.776	-104.388	-14.126	-2.005
ν_{17}	792.227	774.713	0.576	206.070	-14.123	-1.803
ν_{18}	705.310	695.662	4.182	-283.606	7.969	-5.127
ν_{19}	483.230	472.790	8.149	119.721	-31.217	-10.880
ν_{20}	344.228	336.221	5.753	21.270	0.907	-7.144
ν_{21}	194.576	182.295	12.446	420.341	-83.469	-21.569

[a] At the fc-CCSD(T)/cc-pVTZ level.
[b] At the fc-MP2/cc-pVTZ level.

NMR spectra of 2-[(2-chloroethyl)amino]acetonitrile and 2-azabutadiene

^{1}H NMR spectra (400 MHz)

2-[(2-chloroethyl)amino]acetonitrile ($ClCH_2CH_2NHCH_2CN$) in $CDCl_3$

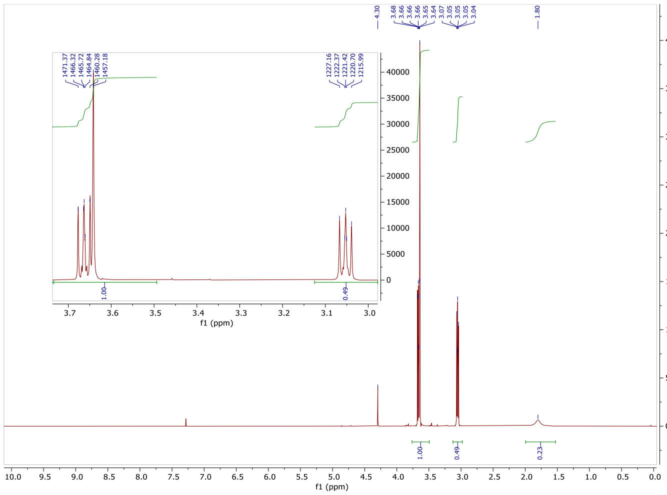

2-azabutadiene ($CH_2{=}N{-}CH{=}CH_2$) in CD_2Cl_2

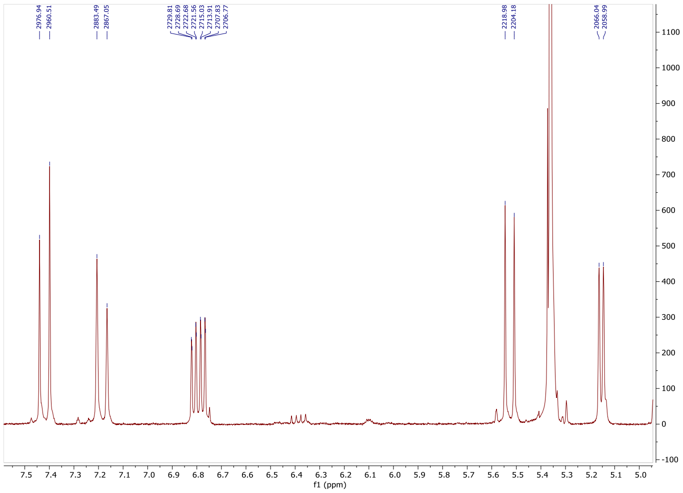

^{13}C NMR spectra (100 MHz)

2-[(2-chloroethyl)amino]acetonitrile ($ClCH_2CH_2NHCH_2CN$) in $CDCl_3$

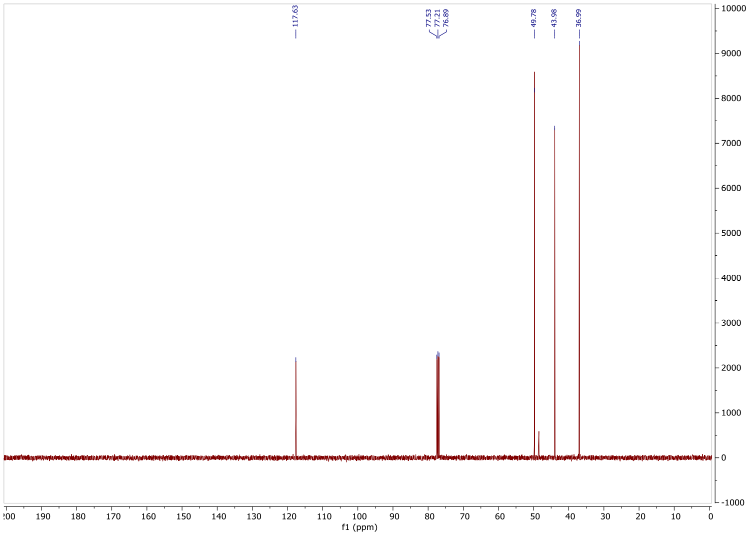

2-azabutadiene ($CH_2{=}N{-}CH{=}CH_2$) in CD_2Cl_2

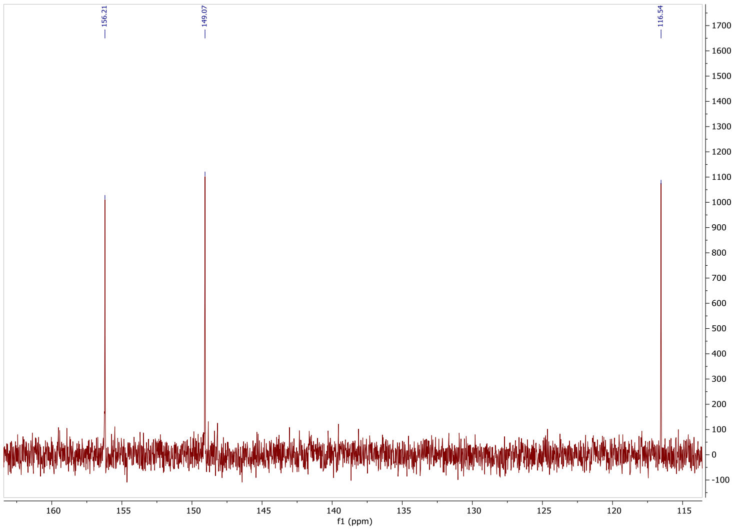

Rotational analysis

Table S6 contains the full list of transitions included in our analysis of *trans*-2-azabutadiene. The first 4 columns are the quantum numbers of the upper state, while the next 4 columns are the quantum numbers of the lower state. The quantum numbers are J, K_a, K_c, and F. The last quantum number is relevant only for transitions with resolved hyperfine structure. In the absence of hyperfine splitting, F is not given. The following columns are (i) the experimental frequency, (ii) the residuals between observed frequencies and those calculated from the final set of parameters, and (iii) in case of blended lines only, the residuals between experimental frequencies and the average frequency of the blended transitions. These last 3 columns are given in units of MHz.

Table S6: List of assigned transitions together with their residuals for *trans*-2-azabutadiene (S reduction, I^r representation).

J'	K'_a	K'_c	F'	J	K_a	K_c	F	Frequency	Obs.-Calc.	Average
1	0	1		0	0	0		9316.690	0.095	
2	0	2		1	0	1		18629.510	0.017	
2	1	1		1	1	0		19089.610	-0.030	
3	0	3		2	0	2		27935.000	0.003	
3	1	2		2	1	1		28632.170	0.081	
3	2	2		2	2	1		27949.900	0.014	
3	2	1		2	2	0		27964.560	-0.020	
4	0	4		3	0	3		37229.390	-0.030	
4	1	4		3	1	3		36346.040	-0.018	
4	1	3		3	1	2		38171.750	0.084	
4	2	3		3	2	2		37263.550	0.002	
4	2	2		3	2	1		37300.200	-0.071	
4	3	2		3	3	1		37274.020	0.000	-0.055
4	3	1		3	3	0		37274.020	-0.111	-0.055
5	0	5		4	0	4		46509.100	0.001	
5	1	5		4	1	4		45425.660	0.007	
5	1	4		4	1	3		47707.440	0.069	
5	2	4		4	2	3		46574.730	0.063	0.063

Continued on next page

Table S6 – *Continued from previous page*

J'	K'_a	K'_c	F'	J	K_a	K_c	F	Frequency	Obs.-Calc.	Average
5	2	4		4	2	3		46574.730	0.063	0.063
12	1	11		12	1	12		35504.870	0.036	
13	1	12		13	1	13		41381.460	-0.002	
22	2	20		22	2	21		33225.320	0.000	
23	2	21		23	2	22		38623.520	-0.015	
1	1	0		1	0	1		42755.610	0.013	
2	1	1		2	0	2		43215.700	-0.044	
3	1	2		3	0	3		43912.760	-0.076	
4	1	3		4	0	4		44855.120	0.038	
5	1	4		5	0	5		46053.310	-0.044	
6	1	5		6	0	6		47521.130	0.039	
7	1	6		7	0	7		49274.160	0.005	
6	0	6		5	1	5		16563.030	0.013	
7	0	7		6	1	6		27072.170	-0.016	
8	0	8		7	1	7		37725.890	0.008	
9	0	9		8	1	8		48501.890	-0.035	
7	2	6		8	1	7		44958.040	0.031	
8	2	7		9	1	8		33654.500	0.027	
9	2	8		10	1	9		22145.880	0.031	
10	2	9		11	1	10		10437.440	0.011	
13	1	12		12	2	11		13553.330	0.003	
14	1	13		13	2	12		25820.440	-0.001	
15	1	14		14	2	13		38256.680	0.006	
9	2	7		10	1	10		48420.250	-0.003	
10	2	8		11	1	11		42304.310	0.022	
11	2	9		12	1	12		36630.750	0.010	
12	2	10		13	1	13		31435.720	0.015	
13	2	11		14	1	14		26755.670	0.010	
14	2	12		15	1	15		22626.500	-0.027	
15	2	13		16	1	16		19082.590	-0.081	
16	2	14		17	1	17		16155.900	-0.034	
17	2	15		18	1	18		13874.750	0.003	
18	2	16		19	1	19		12263.390	-0.002	
19	2	17		20	1	20		11341.460	-0.011	
20	2	18		21	1	21		11123.590	-0.006	
21	2	19		22	1	22		11619.270	-0.022	
22	2	20		23	1	23		12833.120	0.014	

Continued on next page

Table S6 – *Continued from previous page*

J'	K_a'	K_c'	F'	J	K_a	K_c	F	Frequency	Obs.-Calc.	Average
23	2	21		24	1	24		14764.840	-0.016	
24	2	22		25	1	25		17409.900	-0.076	
27	2	25		28	1	28		29522.450	0.016	
28	2	26		29	1	29		34901.380	0.021	
29	2	27		30	1	30		40918.350	-0.011	
30	2	28		31	1	31		47550.050	0.003	
16	3	14		17	2	15		44456.100	0.033	
17	3	15		18	2	16		32925.270	0.002	
19	3	17		20	2	18		8965.770	0.034	
22	2	20		21	3	19		16207.250	-0.020	
23	2	21		22	3	20		29240.420	-0.019	
24	2	22		23	3	21		42560.150	-0.003	
22	3	19		23	2	22		12529.940	0.004	
18	3	15		19	2	18		41978.680	0.032	
19	3	16		20	2	19		34183.040	0.065	
21	3	18		22	2	21		19425.520	0.029	
27	2	26		26	3	23		10976.760	0.011	
30	2	29		29	3	26		23237.050	0.001	
31	2	30		30	3	27		26097.110	0.012	
26	4	22		27	3	25		45318.390	0.024	
27	4	23		28	3	26		36299.780	0.003	
28	4	24		29	3	27		27397.040	0.013	
29	4	25		30	3	28		18630.980	0.020	
30	4	26		31	3	29		10024.440	0.008	
25	4	22		26	3	23		45981.290	0.031	
26	4	23		27	3	24		34846.120	0.013	
27	4	24		28	3	25		23446.020	0.022	
28	4	25		29	3	26		11759.460	-0.004	
31	3	28		30	4	27		12549.990	0.023	
25	2	24		24	2	23		231323.656	0.002	
25	12	14		24	12	13		232993.533	0.020	-0.002
25	12	13		24	12	12		232993.533	0.020	-0.002
25	13	12		24	13	11		232993.533	-0.025	-0.002
25	13	13		24	13	12		232993.533	-0.025	-0.002
25	14	11		24	14	10		232996.368	-0.082	-0.082
25	14	12		24	14	11		232996.368	-0.082	-0.082
25	11	15		24	11	14		232997.153	0.038	0.038

Continued on next page

Table S6 – *Continued from previous page*

J'	K_a'	K_c'	F'	J	K_a	K_c	F	Frequency	Obs.-Calc.	Average
25	11	14		24	11	13		232997.153	0.038	0.038
25	10	15		24	10	14		233005.609	0.020	0.020
25	10	16		24	10	15		233005.609	0.020	0.020
25	16	9		24	16	8		233008.698	-0.077	-0.077
25	16	10		24	16	9		233008.698	-0.077	-0.077
25	9	16		24	9	15		233020.879	-0.010	-0.010
25	9	17		24	9	16		233020.879	-0.010	-0.010
25	8	17		24	8	16		233046.299	-0.003	-0.003
25	8	18		24	8	17		233046.299	-0.003	-0.003
25	7	19		24	7	18		233087.735	0.012	0.012
25	7	18		24	7	17		233087.735	0.012	0.012
25	6	20		24	6	19		233156.636	0.017	-0.011
25	6	19		24	6	18		233156.636	-0.039	-0.011
25	5	21		24	5	20		233276.858	0.005	
25	5	20		24	5	19		233279.738	0.018	
25	3	23		24	3	22		233427.036	-0.040	
25	4	22		24	4	21		233474.922	0.000	
25	4	21		24	4	20		233563.333	0.003	
26	1	26		25	1	25		234334.354	0.013	
26	0	26		25	0	25		235210.335	-0.019	
25	1	24		24	1	23		235470.342	0.003	
25	2	23		24	2	22		237684.259	-0.020	
26	2	25		25	2	24		240446.461	-0.026	
26	13	14		25	13	13		242312.887	-0.011	-0.011
26	13	13		25	13	12		242312.887	-0.011	-0.011
26	12	14		25	12	13		242313.701	0.006	0.006
26	12	15		25	12	14		242313.701	0.006	0.006
26	14	12		25	14	11		242315.206	-0.033	-0.033
26	14	13		25	14	12		242315.206	-0.033	-0.033
26	11	15		25	11	14		242318.538	0.011	0.011
26	11	16		25	11	15		242318.538	0.011	0.011
26	15	11		25	15	10		242320.181	0.074	0.074
26	15	12		25	15	11		242320.181	0.074	0.074
26	10	16		25	10	15		242328.848	0.076	0.076
26	10	17		25	10	16		242328.848	0.076	0.076
26	9	18		25	9	17		242346.675	0.047	0.047
26	9	17		25	9	16		242346.675	0.047	0.047

Continued on next page

Table S6 – *Continued from previous page*

J'	K'_a	K'_c	F'	J	K_a	K_c	F	Frequency	Obs.-Calc.	Average
26	8	19		25	8	18		242375.797	0.001	0.001
26	8	18		25	8	17		242375.797	0.001	0.001
26	7	20		25	7	19		242422.954	0.046	0.045
26	7	19		25	7	18		242422.954	0.045	0.045
26	6	20		25	6	19		242500.859	-0.106	-0.063
26	6	21		25	6	20		242500.859	-0.020	-0.063
26	5	22		25	5	21		242636.244	-0.022	
26	5	21		25	5	20		242640.391	0.041	
26	3	24		25	3	23		242758.805	0.027	
26	4	23		25	4	22		242852.716	0.046	
26	4	22		25	4	21		242968.595	0.003	
27	1	27		26	1	26		243239.278	-0.022	
27	0	27		26	0	26		244017.126	-0.101	
26	3	23		25	3	22		244367.540	0.004	
26	1	25		25	1	24		244572.611	0.050	
26	2	24		25	2	23		247281.906	-0.005	
27	2	26		26	2	25		249555.684	-0.002	
27	13	14		26	13	13		251632.210	0.019	0.019
27	13	15		26	13	14		251632.210	0.019	0.019
27	12	16		26	12	15		251633.932	0.005	0.018
27	12	15		26	12	14		251633.932	0.005	0.018
27	14	14		26	14	13		251633.932	0.031	0.018
27	14	13		26	14	12		251633.932	0.031	0.018
27	15	12		26	15	11		251638.391	0.016	0.016
27	15	13		26	15	12		251638.391	0.016	0.016
27	11	16		26	11	15		251640.087	-0.029	-0.029
27	11	17		26	11	16		251640.087	-0.029	-0.029
27	16	11		26	16	10		251645.137	-0.003	-0.003
27	16	12		26	16	11		251645.137	-0.003	-0.003
27	10	18		26	10	17		251652.305	0.005	0.005
27	10	17		26	10	16		251652.305	0.005	0.005
27	17	10		26	17	9		251653.858	0.003	0.003
27	17	11		26	17	10		251653.858	0.003	0.003
27	18	9		26	18	8		251664.295	0.025	0.025
27	18	10		26	18	9		251664.295	0.025	0.025
27	9	18		26	9	17		251672.949	0.008	0.008
27	9	19		26	9	18		251672.949	0.008	0.008

Continued on next page

Table S6 – *Continued from previous page*

J'	K'_a	K'_c	F'	J	K_a	K_c	F	Frequency	Obs.-Calc.	Average
27	19	9		26	19	8		251676.198	0.000	0.000
27	19	8		26	19	7		251676.198	0.000	0.000
27	8	19		26	8	18		251706.188	0.002	0.002
27	8	20		26	8	19		251706.188	0.002	0.002
27	7	20		26	7	19		251759.467	-0.005	-0.004
27	7	21		26	7	20		251759.467	-0.003	-0.004
27	6	21		26	6	20		251847.325	-0.071	-0.005
27	6	22		26	6	21		251847.325	0.060	-0.005
27	5	23		26	5	22		251998.916	-0.008	
27	5	22		26	5	21		252004.659	-0.004	
27	3	25		26	3	24		252084.107	-0.015	
28	1	28		27	1	27		252138.116	-0.017	
27	4	24		26	4	23		252233.303	-0.011	
27	4	23		26	4	22		252383.619	-0.001	
28	0	28		27	0	27		252825.490	-0.015	
27	1	26		26	1	25		253635.379	-0.011	
27	3	24		26	3	23		253987.816	-0.006	
27	2	25		26	2	24		256858.055	0.013	
28	2	27		27	2	26		258651.140	0.000	
28	13	16		27	13	15		260951.367	-0.065	-0.065
28	13	15		27	13	14		260951.367	-0.065	-0.065
28	12	16		27	12	15		260954.222	0.010	0.010
28	12	17		27	12	16		260954.222	0.010	0.010
28	15	14		27	15	13		260956.458	0.012	0.012
28	15	13		27	15	12		260956.458	0.012	0.012
28	16	13		27	16	12		260962.992	0.042	0.042
28	16	12		27	16	11		260962.992	0.042	0.042
28	17	12		27	17	11		260971.504	-0.061	-0.061
28	17	11		27	17	10		260971.504	-0.061	-0.061
28	10	19		27	10	18		260976.156	-0.032	-0.032
28	10	18		27	10	17		260976.156	-0.032	-0.032
28	18	11		27	18	10		260981.970	-0.041	-0.041
28	18	10		27	18	9		260981.970	-0.041	-0.041
28	9	20		27	9	19		260999.853	0.001	0.001
28	9	19		27	9	18		260999.853	0.001	0.001
29	1	29		28	1	28		261031.266	-0.010	
28	8	20		27	8	19		261037.507	-0.002	-0.002

Continued on next page

Table S6 – *Continued from previous page*

J'	K'_a	K'_c	F'	J	K_a	K_c	F	Frequency	Obs.-Calc.	Average
28	8	21		27	8	20		261037.507	-0.002	-0.002
28	7	22		27	7	21		261097.473	0.013	0.012
28	7	21		27	7	20		261097.473	0.010	0.012
28	6	23		27	6	22		261195.962	0.103	0.005
28	6	22		27	6	21		261195.962	-0.093	0.005
28	5	24		27	5	23		261364.962	0.048	
28	5	23		27	5	22		261372.852	-0.024	
28	3	26		27	3	25		261402.214	0.005	
28	4	25		27	4	24		261616.581	0.030	
28	3	25		27	3	24		263633.445	0.022	
28	2	26		27	2	25		266410.203	0.002	
29	2	28		28	2	27		267732.796	-0.006	
30	1	30		29	1	29		269919.160	0.004	
29	13	16		28	13	15		270270.709	0.088	0.088
29	13	17		28	13	16		270270.709	0.088	0.088
29	12	18		28	12	17		270274.482	-0.068	-0.068
29	12	17		28	12	16		270274.482	-0.068	-0.068
29	11	18		28	11	17		270283.881	0.026	0.026
29	11	19		28	11	18		270283.881	0.026	0.026
29	10	19		28	10	18		270300.461	0.014	0.014
29	10	20		28	10	19		270300.461	0.014	0.014
29	9	20		28	9	19		270327.381	-0.001	-0.001
29	9	21		28	9	20		270327.381	-0.001	-0.001
29	8	21		28	8	20		270369.801	0.002	0.002
29	8	22		28	8	21		270369.801	0.002	0.002
29	7	23		28	7	22		270436.933	-0.001	-0.004
29	7	22		28	7	21		270436.933	-0.006	-0.004
30	0	30		29	0	29		270448.772	-0.015	
29	6	23		28	6	22		270546.904	-0.132	0.012
29	6	24		28	6	23		270546.904	0.157	0.012
29	3	27		28	3	26		270712.166	0.014	
29	5	25		28	5	24		270734.312	-0.004	
29	5	24		28	5	23		270745.232	0.005	
29	4	26		28	4	25		271002.048	0.031	
29	4	25		28	4	24		271247.005	-0.019	
29	3	26		28	3	25		273303.077	-0.007	
29	2	27		28	2	26		275936.148	-0.011	

Continued on next page

Table S6 – *Continued from previous page*

J'	K'_a	K'_c	F'	J	K_a	K_c	F	Frequency	Obs.-Calc.	Average
30	2	29		29	2	28		276800.673	-0.013	
31	1	31		30	1	30		278802.204	0.017	
31	0	31		30	0	30		279264.391	-0.021	
30	13	17		29	13	16		279589.801	0.046	0.046
30	13	18		29	13	17		279589.801	0.046	0.046
30	15	15		29	15	14		279591.977	0.012	0.012
30	15	16		29	15	15		279591.977	0.012	0.012
30	12	19		29	12	18		279594.922	-0.021	-0.021
30	12	18		29	12	17		279594.922	-0.021	-0.021
30	16	14		29	16	13		279597.770	-0.010	-0.010
30	16	15		29	16	14		279597.770	-0.010	-0.010
30	11	19		29	11	18		279606.024	0.007	0.007
30	11	20		29	11	19		279606.024	0.007	0.007
30	18	12		29	18	11		279616.447	0.003	0.003
30	18	13		29	18	12		279616.447	0.003	0.003
30	10	20		29	10	19		279625.104	0.011	0.011
30	10	21		29	10	20		279625.104	0.011	0.011
30	19	12		29	19	11		279628.728	0.030	0.030
30	19	11		29	19	10		279628.728	0.030	0.030
30	20	10		29	20	9		279642.656	0.037	0.037
30	20	11		29	20	10		279642.656	0.037	0.037
30	9	21		29	9	20		279655.574	0.021	0.021
30	9	22		29	9	21		279655.574	0.021	0.021
30	8	22		29	8	21		279703.099	0.007	0.007
30	8	23		29	8	22		279703.099	0.007	0.007
30	7	24		29	7	23		279777.965	0.018	0.014
30	7	23		29	7	22		279777.965	0.010	0.014
30	6	24		29	6	23		279900.161	-0.272	-0.062
30	6	25		29	6	24		279900.161	0.149	-0.062
30	3	28		29	3	27		280013.085	-0.006	
30	5	26		29	5	25		280107.191	-0.002	
30	5	25		29	5	24		280121.979	0.001	
30	4	27		29	4	26		280389.332	0.044	
30	1	29		29	1	28		280590.623	-0.011	
30	4	26		29	4	25		280697.658	-0.008	
30	3	27		29	3	26		282994.829	0.008	
30	2	28		29	2	27		285433.872	-0.001	

Continued on next page

Table S6 – *Continued from previous page*

J'	K'_a	K'_c	F'	J	K_a	K_c	F	Frequency	Obs.-Calc.	Average
32	1	32		31	1	31		287680.798	0.036	
32	0	32		31	0	31		288082.802	0.005	
31	14	18		30	14	17		288907.229	0.053	0.053
31	14	17		30	14	16		288907.229	0.053	0.053
31	13	19		30	13	18		288908.844	0.013	0.013
31	13	18		30	13	17		288908.844	0.013	0.013
31	15	16		30	15	15		288909.432	0.034	0.034
31	15	17		30	15	16		288909.432	0.034	0.034
31	12	19		30	12	18		288915.360	-0.033	-0.033
31	12	20		30	12	19		288915.360	-0.033	-0.033
31	17	14		30	17	13		288922.833	0.023	0.023
31	17	15		30	17	14		288922.833	0.023	0.023
31	11	20		30	11	19		288928.396	0.013	0.013
31	11	21		30	11	20		288928.396	0.013	0.013
31	10	21		30	10	20		288950.137	0.001	0.001
31	10	22		30	10	21		288950.137	0.001	0.001
31	9	23		30	9	22		288984.389	0.003	0.003
31	9	22		30	9	21		288984.389	0.003	0.003
31	8	23		30	8	22		289037.412	-0.009	-0.008
31	8	24		30	8	23		289037.412	-0.008	-0.008
31	7	25		30	7	24		289120.553	0.000	-0.006
31	7	24		30	7	23		289120.553	-0.012	-0.006
31	6	26		30	6	25		289255.824	0.087	
31	6	25		30	6	24		289256.367	0.025	
31	3	29		30	3	28		289304.214	0.021	
31	5	27		30	5	26		289483.557	-0.037	
31	1	30		30	1	29		289502.520	0.064	
31	4	27		30	4	26		290162.608	0.017	
31	3	28		30	3	27		292705.882	-0.034	
32	2	31		31	2	30		294895.465	-0.026	
31	2	29		30	2	28		294901.414	-0.014	
33	1	33		32	1	32		296555.249	-0.003	
33	0	33		32	0	32		296903.846	-0.014	
32	14	18		31	14	17		298225.194	0.068	0.068
32	14	19		31	14	18		298225.194	0.068	0.068
32	15	18		31	15	17		298226.625	0.022	0.022
32	15	17		31	15	16		298226.625	0.022	0.022

Continued on next page

Table S6 – *Continued from previous page*

J'	K'_a	K'_c	F'	J	K_a	K_c	F	Frequency	Obs.-Calc.	Average
32	13	20		31	13	19		298227.871	0.023	0.023
32	13	19		31	13	18		298227.871	0.023	0.023
32	16	16		31	16	15		298231.494	0.004	0.004
32	16	17		31	16	16		298231.494	0.004	0.004
32	12	20		31	12	19		298235.887	-0.014	-0.014
32	12	21		31	12	20		298235.887	-0.014	-0.014
32	17	15		31	17	14		298239.256	0.030	0.030
32	17	16		31	17	15		298239.256	0.030	0.030
32	18	15		31	18	14		298249.385	-0.011	-0.011
32	18	14		31	18	13		298249.385	-0.011	-0.011
32	11	22		31	11	21		298250.942	-0.017	-0.017
32	11	21		31	11	20		298250.942	-0.017	-0.017
32	19	14		31	19	13		298261.720	0.027	0.027
32	19	13		31	19	12		298261.720	0.027	0.027
32	10	22		31	10	21		298275.591	0.000	0.000
32	10	23		31	10	22		298275.591	0.000	0.000
32	9	23		31	9	22		298313.903	0.000	0.000
32	9	24		31	9	23		298313.903	0.000	0.000
32	8	24		31	8	23		298372.821	0.000	0.000
32	8	25		31	8	24		298372.821	0.000	0.000
32	1	31		31	1	30		298381.644	0.033	
32	7	26		31	7	25		298464.808	0.000	-0.009
32	7	25		31	7	24		298464.808	-0.018	-0.009
32	3	30		31	3	29		298584.653	-0.011	
32	6	27		31	6	26		298613.964	-0.042	
32	6	26		31	6	25		298614.924	0.059	
32	5	28		31	5	27		298863.518	-0.032	
32	5	27		31	5	26		298889.856	-0.009	
32	4	29		31	4	28		299167.246	0.022	
32	4	28		31	4	27		299643.107	-0.039	-0.039
32	4	28		31	4	27		299643.107	-0.039	-0.039
32	3	29		31	3	28		302432.952	-0.009	
33	2	32		32	2	31		303922.747	-0.005	
32	2	30		31	2	29		304336.994	0.003	
34	1	34		33	1	33		305426.014	0.008	
34	0	34		33	0	33		305727.429	-0.010	
33	1	32		32	1	31		307231.359	-0.012	

Continued on next page

Table S6 – *Continued from previous page*

J'	K_a'	K_c'	F'	J	K_a	K_c	F	Frequency	Obs.-Calc.	Average
33	14	19		32	14	18		307542.970	0.052	0.052
33	14	20		32	14	19		307542.970	0.052	0.052
33	13	20		32	13	19		307546.720	-0.083	-0.083
33	13	21		32	13	20		307546.720	-0.083	-0.083
33	17	16		32	17	15		307555.269	-0.021	-0.021
33	17	17		32	17	16		307555.269	-0.021	-0.021
33	12	21		32	12	20		307556.479	0.010	0.010
33	12	22		32	12	21		307556.479	0.010	0.010
33	18	15		32	18	14		307565.222	-0.063	-0.063
33	18	16		32	18	15		307565.222	-0.063	-0.063
33	11	22		32	11	21		307573.776	0.024	0.024
33	11	23		32	11	22		307573.776	0.024	0.024
33	20	14		32	20	13		307591.785	-0.039	-0.039
33	20	13		32	20	12		307591.785	-0.039	-0.039
33	10	23		32	10	22		307601.471	0.002	0.002
33	10	24		32	10	23		307601.471	0.002	0.002
33	9	25		32	9	24		307644.137	0.010	0.010
33	9	24		32	9	23		307644.137	0.010	0.010
33	8	25		32	8	24		307709.341	0.011	0.012
33	8	26		32	8	25		307709.341	0.012	0.012
33	7	26		32	7	25		307810.780	-0.014	-0.001
33	7	27		32	7	26		307810.780	0.013	-0.001
33	3	31		32	3	30		307853.756	0.004	
33	6	28		32	6	27		307974.883	-0.019	
33	6	27		32	6	26		307976.128	0.021	
33	5	28		32	5	27		308281.727	0.049	
33	4	30		32	4	29		308556.647	-0.083	-0.083
33	4	30		32	4	29		308556.647	-0.083	-0.083
3	3	1		2	2	0		240565.232	-0.027	
3	3	0		2	2	1		240568.921	-0.031	
4	3	2		3	2	1		249874.680	-0.019	
4	3	1		3	2	2		249893.204	0.007	
4	4	1		4	3	2		297628.441	-0.032	0.033
4	4	0		4	3	1		297628.441	0.097	0.033
5	3	3		4	2	2		259170.071	-0.026	
5	3	2		4	2	3		259225.711	0.006	
6	4	2		7	3	5		232372.985	-0.015	

Continued on next page

Table S6 – *Continued from previous page*

J'	K'_a	K'_c	F'	J	K_a	K_c	F	Frequency	Obs.-Calc.	Average
6	3	4		5	2	3		268441.328	-0.013	
6	3	3		5	2	4		268571.384	0.001	
6	4	3		6	3	4		297618.263	0.020	
7	3	5		6	2	4		277675.677	0.003	
7	3	4		6	2	5		277936.315	-0.033	
7	4	3		7	3	4		297604.060	-0.009	
7	4	4		7	3	5		297607.927	-0.008	
8	3	6		7	2	5		286857.735	-0.004	
8	3	5		7	2	6		287327.969	-0.015	
8	4	4		8	3	5		297584.356	0.000	
8	4	5		8	3	6		297592.840	-0.008	
9	3	7		8	2	6		295969.701	-0.001	
9	3	6		8	2	7		296755.007	0.002	
9	4	5		9	3	6		297555.061	-0.005	
9	4	6		9	3	7		297572.018	-0.001	
10	5	6		11	4	7		280039.245	0.093	
10	5	5		11	4	8		280039.552	-0.096	
10	4	6		10	3	7		297513.163	0.000	
10	4	7		10	3	8		297544.558	-0.023	
10	3	8		9	2	7		304991.463	0.016	
10	3	7		9	2	8		306227.513	-0.017	
11	5	7		12	4	8		270687.971	-0.022	
11	5	6		12	4	9		270689.042	0.056	
11	4	7		11	3	8		297454.979	-0.010	
11	4	8		11	3	9		297509.822	-0.011	
12	5	7		13	4	10		261329.908	-0.004	
12	4	8		12	3	9		297376.164	-0.007	
12	4	9		12	3	10		297467.299	-0.015	
13	5	9		14	4	10		251957.819	-0.010	
13	5	8		14	4	11		251961.198	-0.007	
13	4	9		13	3	10		297271.535	0.003	
13	4	10		13	3	11		297416.871	-0.008	
14	5	10		15	4	11		242575.778	0.010	
14	5	9		15	4	12		242581.632	0.031	
14	4	10		14	3	11		297135.002	0.005	
14	4	11		14	3	12		297358.768	-0.003	
15	5	11		16	4	12		233180.095	0.013	

Continued on next page

Table S6 – *Continued from previous page*

J'	K_a'	K_c'	F'	J	K_a	K_c	F	Frequency	Obs.-Calc.	Average
15	5	10		16	4	13		233189.794	-0.010	
15	4	11		15	3	12		296959.498	0.006	
15	4	12		15	3	13		297293.688	-0.012	
16	4	12		16	3	13		296736.849	-0.012	
16	4	13		16	3	14		297222.916	0.005	
17	4	13		17	3	14		296457.776	-0.004	
17	4	14		17	3	15		297148.252	0.001	
18	6	13		19	5	14		290183.019	0.061	
18	4	14		18	3	15		296111.709	0.013	
18	4	15		18	3	16		297072.233	0.004	
19	6	14		20	5	15		280788.772	-0.008	
19	6	13		20	5	16		280789.809	0.079	
19	4	15		19	3	16		295686.780	-0.006	
20	6	14		21	5	17		271384.413	0.001	
20	4	16		20	3	17		295169.972	0.013	
20	4	17		20	3	18		296929.731	0.004	
21	4	17		21	3	18		294546.905	0.000	
21	4	18		21	3	19		296871.968	0.002	
22	6	17		23	5	18		252531.096	0.000	
22	6	16		23	5	19		252534.902	-0.005	
22	4	18		22	3	19		293802.230	0.025	
22	4	19		22	3	20		296830.325	0.000	
23	6	18		24	5	19		243082.667	0.001	
23	6	17		24	5	20		243088.482	0.003	
23	4	19		23	3	20		292919.538	0.013	
23	4	20		23	3	21		296811.142	0.002	
24	6	19		25	5	20		233617.369	0.022	
24	6	18		25	5	21		233626.061	-0.001	
24	4	20		24	3	21		291881.925	0.032	
25	1	25		24	0	24		233161.716	0.020	
25	4	21		25	3	22		290672.120	0.029	
25	4	22		25	3	23		296869.380	0.002	
26	1	26		25	0	25		241092.202	0.010	
27	0	27		26	1	26		238135.393	0.005	
27	1	27		26	0	26		249121.136	-0.003	
27	7	21		28	6	22		290681.567	0.034	
27	7	20		28	6	23		290682.025	-0.070	

Continued on next page

Table S6 – *Continued from previous page*

J'	K_a'	K_c'	F'	J	K_a	K_c	F	Frequency	Obs.-Calc.	Average
27	4	24		27	3	25		297112.459	-0.002	
28	0	28		27	1	27		247721.571	-0.022	
28	1	28		27	0	27		257242.041	-0.003	
28	7	22		29	6	23		281231.928	-0.029	
28	7	21		29	6	24		281232.846	0.035	
28	4	25		28	3	26		297326.791	-0.013	
29	0	29		28	1	28		257219.336	-0.008	
29	1	29		28	0	28		265447.833	0.017	
29	4	26		29	3	27		297616.686	0.017	
30	7	23		31	6	26		262291.992	0.036	
30	0	30		29	1	29		266636.846	-0.009	
30	1	30		29	0	29		273731.092	0.005	
30	4	27		30	3	28		297992.880	0.014	
31	7	25		32	6	26		252795.783	0.032	
31	7	24		32	6	27		252798.539	0.024	
31	0	31		30	1	30		275982.068	-0.042	
31	1	31		30	0	30		282084.476	-0.012	
32	7	26		33	6	27		243284.451	-0.001	
32	7	25		33	6	28		243288.403	-0.036	
32	0	32		31	1	31		285262.714	-0.006	
32	1	32		31	0	31		290500.844	0.006	
33	7	27		34	6	28		233755.033	-0.007	
33	7	26		34	6	29		233760.747	0.018	
33	0	33		32	1	32		294485.828	0.009	
34	0	34		33	1	33		303658.007	0.001	
34	1	34		33	0	33		307495.454	0.015	
35	4	32		35	3	33		301565.532	0.004	
35	0	35		34	1	34		312785.344	0.025	
35	1	35		34	0	34		316061.363	0.018	
36	8	29		37	7	30		290927.791	0.238	0.040
36	8	28		37	7	31		290927.791	-0.158	0.040
36	4	33		36	3	34		302698.823	-0.012	
36	0	36		35	1	35		321873.241	0.012	
36	1	36		35	0	35		324665.604	0.013	
37	8	30		38	7	31		281426.447	0.038	
37	8	29		38	7	32		281426.977	-0.006	
37	4	34		37	3	35		303998.020	-0.015	

Continued on next page

Table S6 – *Continued from previous page*

J'	K_a'	K_c'	F'	J	K_a	K_c	F	Frequency	Obs.-Calc.	Average
37	0	37		36	1	36		330926.611	-0.043	
38	8	30		39	7	33		271911.136	-0.078	
38	4	35		38	3	36		305473.813	0.001	
38	0	38		37	1	37		339949.941	-0.042	
39	8	32		40	7	33		262378.762	0.073	
39	8	31		40	7	34		262379.790	-0.070	
39	4	36		39	3	37		307136.396	0.013	
40	8	33		41	7	34		252830.440	-0.023	
40	8	32		41	7	35		252832.131	0.018	
41	8	33		42	7	36		243267.046	-0.098	
42	8	35		43	7	36		233680.911	0.003	
42	8	34		43	7	37		233684.156	0.052	
42	4	39		42	3	40		313338.203	-0.001	
43	4	40		43	3	41		315837.736	-0.084	
45	9	37		46	8	38		290985.575	0.128	0.010
45	9	36		46	8	39		290985.575	-0.107	0.010
46	9	38		47	8	39		281439.916	0.151	-0.014
46	9	37		47	8	40		281439.916	-0.180	-0.014
49	9	41		50	8	42		252709.327	-0.024	
49	9	40		50	8	43		252710.293	0.059	
50	9	42		51	8	43		243099.076	-0.011	
50	9	41		51	8	44		243100.313	0.016	
51	9	43		52	8	44		233470.809	-0.060	
51	9	42		52	8	45		233472.501	-0.013	
54	10	45		55	9	46		290916.199	0.057	-0.007
54	10	44		55	9	47		290916.199	-0.070	-0.007
55	10	46		56	9	47		281333.976	0.114	0.027
55	10	45		56	9	48		281333.976	-0.060	0.027
58	10	49		59	9	50		252495.392	0.139	-0.081
58	10	48		59	9	51		252495.392	-0.302	-0.081
63	5	58		63	4	59		272565.643	0.039	
65	5	60		65	4	61		267513.112	0.025	
66	5	61		66	4	62		265832.688	-0.005	
67	5	62		67	4	63		264771.713	0.015	
68	5	63		68	4	64		264366.191	-0.001	
69	5	64		69	4	65		264647.750	0.022	
70	5	65		70	4	66		265643.279	0.003	

Continued on next page

Table S6 – *Continued from previous page*

J'	K'_a	K'_c	F'	J	K_a	K_c	F	Frequency	Obs.-Calc.	Average
71	5	66		71	4	67		267375.229	0.027	
73	5	68		73	4	69		273114.308	0.044	
25	3	22		24	3	21		234773.132	0.000	
5	4	2		6	3	3		241703.854	-0.049	
64	5	59		64	4	60		269772.379	0.042	
8	5	3		9	4	6		298720.269	-0.047	
33	1	33		32	0	32		298973.284	-0.010	
32	4	29		32	3	30		299049.095	-0.011	
33	4	30		33	3	31		299752.081	-0.003	
26	7	19		27	6	22		300118.308	-0.175	0.006
26	7	20		27	6	21		300118.308	0.188	0.006
35	8	27		36	7	30		300414.745	-0.129	0.006
35	8	28		36	7	29		300414.745	0.141	0.006
53	10	43		54	9	46		300484.193	0.028	0.073
53	10	44		54	9	45		300484.193	0.119	0.073
44	9	35		45	8	38		300516.785	-0.064	0.019
44	9	36		45	8	37		300516.785	0.102	0.019
34	4	31		34	3	32		300587.055	-0.001	
19	4	16		19	3	17		296998.094	0.029	
40	4	37		40	3	38		308995.402	-0.006	
41	4	38		41	3	39		311059.898	-0.013	
10	2	8	9	9	1	9	8	232826.767	0.120	0.014
10	2	8	11	9	1	9	10	232826.767	-0.091	0.014
10	2	8	10	9	1	9	9	232828.762	-0.056	
11	2	9	10	10	1	10	9	245228.086	0.065	-0.035
11	2	9	12	10	1	10	11	245228.086	-0.135	-0.035
12	2	10	11	11	1	11	10	258090.874	0.122	0.027
12	2	10	13	11	1	11	12	258090.874	-0.069	0.027
13	2	12	13	12	1	11	12	231058.739	0.026	
13	2	12	12	12	1	11	11	231060.265	-0.086	-0.022
13	2	12	14	12	1	11	13	231060.265	0.042	-0.022
13	2	11	13	12	1	12	12	271453.043	-0.049	
14	2	13	14	13	1	12	13	237498.142	0.073	
14	2	13	13	13	1	12	12	237499.623	-0.063	-0.004
14	2	13	15	13	1	12	14	237499.623	0.054	-0.004
14	2	12	13	13	1	13	12	285343.171	0.094	0.005
14	2	12	15	13	1	13	14	285343.171	-0.084	0.005

Continued on next page

Table S6 – *Continued from previous page*

J'	K'_a	K'_c	F'	J	K_a	K_c	F	Frequency	Obs.-Calc.	Average
14	2	12	14	13	1	13	13	285345.601	-0.038	
16	2	15	16	15	1	14	15	249798.958	0.034	
16	2	15	15	15	1	14	14	249800.443	-0.052	-0.003
16	2	15	17	15	1	14	16	249800.443	0.047	-0.003
17	2	16	17	16	1	15	16	255678.098	0.013	
17	2	16	16	16	1	15	15	255679.571	-0.061	-0.015
17	2	16	18	16	1	15	17	255679.571	0.031	-0.015
19	2	18	19	18	1	17	18	266948.851	0.018	
19	2	18	18	18	1	17	17	266950.287	-0.038	0.001
19	2	18	20	18	1	17	19	266950.287	0.041	0.001
20	2	19	20	19	1	18	19	272367.223	0.013	
20	2	19	19	19	1	18	18	272368.632	-0.039	-0.002
20	2	19	21	19	1	18	20	272368.632	0.035	-0.002
21	2	20	21	20	1	19	20	277661.686	0.008	
21	2	20	20	20	1	19	19	277663.030	-0.073	-0.039
21	2	20	22	20	1	19	21	277663.030	-0.005	-0.039
22	2	21	22	21	1	20	21	282850.032	-0.007	
22	2	21	21	21	1	20	20	282851.379	-0.049	-0.017
22	2	21	23	21	1	20	22	282851.379	0.015	-0.017
23	2	22	23	22	1	21	22	287951.906	0.011	
23	2	22	22	22	1	21	21	287953.198	-0.045	-0.015
23	2	22	24	22	1	21	23	287953.198	0.014	-0.015
24	2	23	24	23	1	22	23	292988.582	0.026	
24	2	23	23	23	1	22	22	292989.833	-0.027	0.000
24	2	23	25	23	1	22	24	292989.833	0.027	0.000
25	2	24	25	24	1	23	24	297982.878	-0.012	
25	2	24	26	24	1	23	25	297984.121	0.024	-0.001
25	2	24	24	24	1	23	23	297984.121	-0.026	-0.001
26	3	24	27	26	2	25	27	230449.367	-0.029	-0.017
26	3	24	25	26	2	25	25	230449.367	-0.005	-0.017
26	3	24	26	26	2	25	26	230450.101	0.083	
26	4	22	27	26	3	23	27	289273.228	-0.049	-0.057
26	4	22	25	26	3	23	25	289273.228	-0.064	-0.057
26	4	23	27	26	3	24	27	296963.285	0.021	0.022
26	4	23	25	26	3	24	25	296963.285	0.022	0.022
27	3	25	28	27	2	26	28	232977.782	-0.039	-0.027
27	3	25	26	27	2	26	26	232977.782	-0.014	-0.027

Continued on next page

Table S6 – *Continued from previous page*

J'	K_a'	K_c'	F'	J	K_a	K_c	F	Frequency	Obs.-Calc.	Average
27	3	25	27	27	2	26	27	232978.555	0.077	
27	4	23	28	27	3	24	28	287669.061	-0.026	-0.034
27	4	23	26	27	3	24	26	287669.061	-0.042	-0.034
27	2	26	27	26	1	25	26	307942.230	0.001	
27	2	26	26	26	1	25	25	307943.363	-0.019	0.002
27	2	26	28	26	1	25	27	307943.363	0.023	0.002
28	3	26	29	28	2	27	29	235728.840	-0.039	-0.026
28	3	26	27	28	2	27	27	235728.840	-0.014	-0.026
28	3	26	28	28	2	27	28	235729.629	0.061	
28	4	24	29	28	3	25	29	285845.030	-0.049	-0.057
28	4	24	27	28	3	25	27	285845.030	-0.066	-0.057
28	2	27	28	27	1	26	27	312958.020	0.005	
28	2	27	27	27	1	26	26	312959.098	-0.014	0.005
28	2	27	29	27	1	26	28	312959.098	0.025	0.005
29	2	28	30	29	1	29	30	229872.057	-0.010	0.017
29	2	28	28	29	1	29	28	229872.057	0.045	0.017
29	2	28	29	29	1	29	29	229873.668	0.004	
29	3	27	30	29	2	28	30	238708.205	-0.013	0.000
29	3	27	28	29	2	28	28	238708.205	0.012	0.000
29	4	25	30	29	3	26	30	283789.001	-0.031	-0.040
29	4	25	28	29	3	26	28	283789.001	-0.049	-0.040
30	1	29	29	29	2	28	28	234201.052	0.009	-0.005
30	1	29	31	29	2	28	30	234201.052	-0.019	-0.005
30	1	29	30	29	2	28	29	234201.951	0.068	
30	2	29	31	30	1	30	31	236753.578	-0.023	0.004
30	2	29	29	30	1	30	29	236753.578	0.030	0.004
30	2	29	30	30	1	30	30	236755.196	0.010	
30	3	28	31	30	2	29	31	241920.602	-0.010	0.002
30	3	28	29	30	2	29	29	241920.602	0.015	0.002
30	3	28	30	30	2	29	30	241921.434	0.070	
30	4	26	31	30	3	27	31	281491.913	0.024	0.014
30	4	26	29	30	3	27	29	281491.913	0.005	0.014
31	2	30	32	31	1	31	32	243806.278	-0.009	0.016
31	2	30	30	31	1	31	30	243806.278	0.042	0.016
31	2	30	31	31	1	31	31	243807.844	-0.015	
31	1	30	30	30	2	29	29	246902.821	-0.014	-0.027
31	1	30	32	30	2	29	31	246902.821	-0.039	-0.027

Continued on next page

Table S6 – *Continued from previous page*

J'	K_a'	K_c'	F'	J	K_a	K_c	F	Frequency	Obs.-Calc.	Average
31	1	30	31	30	2	29	30	246903.614	0.002	
31	4	27	31	31	3	28	31	278947.942	-0.039	
31	4	27	32	31	3	28	32	278948.580	0.003	-0.006
31	4	27	30	31	3	28	30	278948.580	-0.016	-0.006
32	3	30	33	32	2	31	33	249059.053	-0.036	-0.024
32	3	30	31	32	2	31	31	249059.053	-0.011	-0.024
32	3	30	32	32	2	31	32	249059.947	0.048	
32	2	31	33	32	1	32	33	251020.985	-0.036	-0.011
32	2	31	31	32	1	32	31	251020.985	0.013	-0.011
32	2	31	32	32	1	32	32	251022.568	-0.011	
32	1	31	31	31	2	30	30	259429.579	-0.019	-0.030
32	1	31	33	31	2	30	32	259429.579	-0.041	-0.030
32	1	31	32	31	2	30	31	259430.386	0.074	
32	4	28	32	32	3	29	32	276158.084	-0.059	
32	4	28	33	32	3	29	33	276158.770	-0.004	-0.013
32	4	28	31	32	3	29	31	276158.770	-0.023	-0.013
33	3	31	34	33	2	32	34	252990.057	-0.023	-0.011
33	3	31	32	33	2	32	32	252990.057	0.002	-0.011
33	3	31	33	33	2	32	33	252990.937	0.020	
33	2	32	34	33	1	33	34	258388.516	-0.009	0.015
33	2	32	32	33	1	33	32	258388.516	0.038	0.015
33	2	32	33	33	1	33	33	258390.065	-0.004	
33	4	29	33	33	3	30	33	273126.839	-0.069	
33	4	29	34	33	3	30	34	273127.605	0.037	0.027
33	4	29	32	33	3	30	32	273127.605	0.017	0.027
34	1	33	35	34	0	34	35	238628.771	0.019	0.050
34	1	33	33	34	0	34	33	238628.771	0.081	0.050
34	3	32	35	34	2	33	35	257163.890	-0.024	-0.011
34	3	32	33	34	2	33	33	257163.890	0.001	-0.011
34	3	32	34	34	2	33	34	257164.817	0.042	
34	2	33	35	34	1	34	35	265899.437	0.003	0.026
34	2	33	33	34	1	34	33	265899.437	0.048	0.026
34	2	33	34	34	1	34	34	265900.944	-0.018	
34	4	30	34	34	3	31	34	269865.223	-0.062	
34	4	30	35	34	3	31	35	269866.008	0.037	0.026
34	4	30	33	34	3	31	33	269866.008	0.016	0.026
34	1	33	33	33	2	32	32	283898.205	0.000	-0.008

Continued on next page

Table S6 – *Continued from previous page*

J'	K_a'	K_c'	F'	J	K_a	K_c	F	Frequency	Obs.-Calc.	Average
34	1	33	35	33	2	32	34	283898.205	-0.017	-0.008
34	1	33	34	33	2	32	33	283898.808	0.014	
35	1	34	36	35	0	35	36	248933.176	-0.046	-0.017
35	1	34	34	35	0	35	34	248933.176	0.012	-0.017
35	1	34	35	35	0	35	35	248935.207	-0.033	
35	3	33	36	35	2	34	36	261580.584	-0.052	-0.040
35	3	33	34	35	2	34	34	261580.584	-0.027	-0.040
35	3	33	35	35	2	34	35	261581.558	0.036	
35	4	31	35	35	3	32	35	266390.451	-0.068	
35	4	31	36	35	3	32	36	266391.271	0.045	0.035
35	4	31	34	35	3	32	34	266391.271	0.025	0.035
35	2	34	36	35	1	35	36	273544.353	-0.022	0.000
35	2	34	34	35	1	35	34	273544.353	0.022	0.000
35	2	34	35	35	1	35	35	273545.848	-0.038	
35	1	34	34	34	2	33	33	295819.142	0.059	0.051
35	1	34	36	34	2	33	35	295819.142	0.043	0.051
36	1	35	37	36	0	36	37	259194.402	-0.006	0.022
36	1	35	35	36	0	36	35	259194.402	0.049	0.022
36	1	35	36	36	0	36	36	259196.333	-0.028	
36	4	32	36	36	3	33	36	262726.132	-0.054	
36	4	32	37	36	3	33	37	262726.947	0.038	0.028
36	4	32	35	36	3	33	35	262726.947	0.018	0.028
36	3	34	37	36	2	35	37	266239.310	-0.026	-0.013
36	3	34	35	36	2	35	35	266239.310	-0.001	-0.013
36	2	35	37	36	1	36	37	281314.040	-0.002	0.019
36	2	35	35	36	1	36	35	281314.040	0.040	0.019
36	2	35	36	36	1	36	36	281315.515	-0.021	
36	1	35	35	35	2	34	34	307523.306	0.063	0.056
36	1	35	37	35	2	34	36	307523.306	0.050	0.056
37	2	35	36	36	3	34	35	234097.294	-0.001	-0.017
37	2	35	38	36	3	34	37	234097.294	-0.032	-0.017
37	2	35	37	36	3	34	36	234098.503	0.044	
37	4	33	37	37	3	34	37	258902.014	-0.069	
37	4	33	38	37	3	34	38	258902.858	0.046	0.036
37	4	33	36	37	3	34	36	258902.858	0.026	0.036
37	1	36	38	37	0	37	38	269396.802	0.005	0.031
37	1	36	36	37	0	37	36	269396.802	0.056	0.031

Continued on next page

Table S6 – *Continued from previous page*

J'	K_a'	K_c'	F'	J	K_a	K_c	F	Frequency	Obs.-Calc.	Average
37	1	36	37	37	0	37	37	269398.662	-0.026	
37	3	35	38	37	2	36	38	271138.109	-0.045	-0.033
37	3	35	36	37	2	36	36	271138.109	-0.021	-0.033
37	2	36	38	37	1	37	38	289199.295	0.025	0.045
37	2	36	36	37	1	37	36	289199.295	0.065	0.045
37	2	36	37	37	1	37	37	289200.741	-0.004	
37	1	36	36	36	2	35	35	319009.503	0.109	0.103
37	1	36	38	36	2	35	37	319009.503	0.097	0.103
38	2	36	37	37	3	35	36	249476.420	0.005	-0.009
38	2	36	39	37	3	35	38	249476.420	-0.024	-0.009
38	2	36	38	37	3	35	37	249477.544	-0.001	
38	4	34	38	38	3	35	38	254953.833	-0.055	
38	4	34	39	38	3	35	39	254954.668	0.050	0.040
38	4	34	37	38	3	35	37	254954.668	0.031	0.040
38	3	36	39	38	2	37	39	276274.284	-0.022	-0.009
38	3	36	37	38	2	37	37	276274.284	0.003	-0.009
38	3	36	38	38	2	37	38	276275.308	0.054	
38	1	37	39	38	0	38	39	279528.884	0.013	0.037
38	1	37	37	38	0	38	37	279528.884	0.061	0.037
38	1	37	38	38	0	38	38	279530.688	-0.012	
38	2	37	39	38	1	38	39	297191.118	0.017	0.036
38	2	37	37	38	1	38	37	297191.118	0.055	0.036
38	2	37	38	38	1	38	38	297192.553	-0.005	
39	4	35	39	39	3	36	39	250922.592	-0.058	
39	4	35	40	39	3	36	40	250923.418	0.045	0.036
39	4	35	38	39	3	36	38	250923.418	0.027	0.036
39	2	37	38	38	3	36	37	264817.876	-0.003	-0.016
39	2	37	40	38	3	36	39	264817.876	-0.030	-0.016
39	2	37	39	38	3	36	38	264818.975	0.005	
39	3	37	40	39	2	38	40	281644.113	-0.004	0.009
39	3	37	38	39	2	38	38	281644.113	0.021	0.009
39	3	37	39	39	2	38	39	281645.117	0.035	
39	1	38	40	39	0	39	40	289582.717	-0.028	-0.005
39	1	38	38	39	0	39	38	289582.717	0.018	-0.005
39	1	38	39	39	0	39	39	289584.492	-0.022	
39	2	38	40	39	1	39	40	305280.855	0.013	0.032
39	2	38	38	39	1	39	38	305280.855	0.050	0.032

Continued on next page

Table S6 – *Continued from previous page*

J'	K'_a	K'_c	F'	J	K_a	K_c	F	Frequency	Obs.-Calc.	Average
39	2	38	39	39	1	39	39	305282.285	0.006	
40	4	36	40	40	3	37	40	246854.040	-0.069	
40	4	36	41	40	3	37	41	246854.876	0.061	0.052
40	4	36	39	40	3	37	39	246854.876	0.043	0.052
40	3	38	41	40	2	39	41	287243.063	0.009	0.021
40	3	38	39	40	2	39	39	287243.063	0.033	0.021
40	3	38	40	40	2	39	40	287244.033	-0.002	
40	2	38	41	39	3	37	40	280092.618	-0.015	-0.002
40	2	38	39	39	3	37	38	280092.618	0.010	-0.002
40	2	38	40	39	3	37	39	280093.668	0.009	
41	4	37	41	41	3	38	41	242797.820	-0.067	
41	4	37	40	41	3	38	40	242798.609	0.022	
41	4	37	42	41	3	38	42	242798.613	0.043	
41	3	39	42	41	2	40	42	293065.768	-0.017	-0.005
41	3	39	40	41	2	40	40	293065.768	0.007	-0.005
41	3	39	41	41	2	40	41	293066.776	-0.004	
41	2	39	40	40	3	38	39	295271.368	-0.008	-0.020
41	2	39	42	40	3	38	41	295271.368	-0.032	-0.020
41	2	39	41	40	3	38	40	295272.402	0.018	
41	1	40	42	41	0	41	42	309439.797	-0.032	-0.011
41	1	40	40	41	0	41	40	309439.797	0.009	-0.011
41	1	40	41	41	0	41	41	309441.530	0.044	
42	2	40	43	42	1	41	43	230057.052	-0.001	0.021
42	2	40	41	42	1	41	41	230057.052	0.042	0.021
42	2	40	42	42	1	41	42	230058.741	-0.039	
42	4	38	42	42	3	39	42	238806.605	0.007	
42	4	38	43	42	3	39	43	238807.256	0.005	-0.003
42	4	38	41	42	3	39	41	238807.256	-0.010	-0.003
42	2	40	41	41	3	39	40	310325.166	-0.040	-0.051
42	2	40	43	41	3	39	42	310325.166	-0.063	-0.051
43	4	39	43	43	3	40	43	234934.895	-0.001	
43	4	39	44	43	3	40	44	234935.534	0.023	0.016
43	4	39	42	43	3	40	42	234935.534	0.009	0.016
43	2	41	44	43	1	42	44	240691.343	-0.027	-0.007
43	2	41	42	43	1	42	42	240691.343	0.013	-0.007
43	2	41	43	43	1	42	43	240693.085	-0.009	
43	3	41	44	43	2	42	44	305357.591	-0.016	-0.004

Continued on next page

Table S6 – *Continued from previous page*

J'	K'_a	K'_c	F'	J	K_a	K_c	F	Frequency	Obs.-Calc.	Average
43	3	41	42	43	2	42	42	305357.591	0.008	-0.004
43	3	41	43	43	2	42	43	305358.655	0.030	
44	4	40	44	44	3	41	44	231238.412	-0.084	
44	4	40	45	44	3	41	45	231239.086	0.021	0.014
44	4	40	43	44	3	41	43	231239.086	0.008	0.014
44	2	42	45	44	1	43	45	251515.373	-0.003	0.017
44	2	42	43	44	1	43	43	251515.373	0.036	0.017
44	2	42	44	44	1	43	44	251517.076	-0.012	
44	3	42	45	44	2	43	45	311812.478	-0.066	-0.054
44	3	42	43	44	2	43	43	311812.478	-0.043	-0.054
44	3	42	44	44	2	43	44	311813.611	0.040	
45	2	43	46	45	1	44	46	262483.769	-0.063	-0.044
45	2	43	44	45	1	44	44	262483.769	-0.025	-0.044
45	2	43	45	45	1	44	45	262485.441	-0.085	
45	4	42	46	45	3	43	46	321526.728	-0.040	-0.034
45	4	42	44	45	3	43	44	321526.728	-0.029	-0.034
46	3	43	45	45	4	42	44	237150.953	-0.047	-0.059
46	3	43	47	45	4	42	46	237150.953	-0.072	-0.059
46	3	43	46	45	4	42	45	237152.238	0.064	
46	2	44	47	46	1	45	47	273553.439	0.006	0.024
46	2	44	45	46	1	45	45	273553.439	0.042	0.024
46	2	44	46	46	1	45	46	273555.079	-0.024	
47	3	44	46	46	4	43	45	254160.193	-0.045	-0.057
47	3	44	48	46	4	43	47	254160.193	-0.069	-0.057
47	3	44	47	46	4	43	46	254161.377	-0.030	
47	2	45	48	47	1	46	48	284683.648	-0.003	0.014
47	2	45	46	47	1	46	46	284683.648	0.032	0.014
47	2	45	47	47	1	46	47	284685.269	-0.025	
48	3	45	47	47	4	44	46	271286.612	0.033	0.022
48	3	45	49	47	4	44	48	271286.612	0.010	0.022
48	2	46	49	48	1	47	49	295837.459	0.002	0.019
48	2	46	47	48	1	47	47	295837.459	0.036	0.019
48	2	46	48	48	1	47	48	295839.036	-0.031	
49	3	46	48	48	4	45	47	288499.736	0.002	-0.009
49	3	46	50	48	4	45	49	288499.736	-0.021	-0.009
49	3	46	49	48	4	45	48	288500.870	-0.011	
49	2	47	50	49	1	48	50	306981.862	-0.011	0.006

Continued on next page

Table S6 – *Continued from previous page*

J'	K'_a	K'_c	F'	J	K_a	K_c	F	Frequency	Obs.-Calc.	Average
49	2	47	48	49	1	48	48	306981.862	0.022	0.006
49	2	47	49	49	1	48	49	306983.426	-0.021	
50	3	47	49	49	4	46	48	305768.568	-0.072	
50	3	47	51	49	4	46	50	305768.617	-0.045	
52	3	49	53	52	2	50	53	235945.166	-0.025	-0.013
52	3	49	51	52	2	50	51	235945.166	-0.001	-0.013
52	3	49	52	52	2	50	52	235946.469	0.031	
53	3	50	54	53	2	51	54	245532.131	-0.036	-0.024
53	3	50	52	53	2	51	52	245532.131	-0.012	-0.024
53	3	50	53	53	2	51	53	245533.458	0.005	
54	3	51	55	54	2	52	55	255574.021	0.010	0.022
54	3	51	53	54	2	52	53	255574.021	0.034	0.022
54	3	51	54	54	2	52	54	255575.245	-0.086	
54	5	49	55	54	4	50	55	312780.342	0.011	0.004
54	5	49	53	54	4	50	53	312780.342	-0.002	0.004
55	3	52	56	55	2	53	56	266025.795	-0.015	-0.003
55	3	52	54	55	2	53	54	266025.795	0.010	-0.003
55	3	52	55	55	2	53	55	266027.135	-0.020	
55	5	50	55	55	4	51	55	307671.844	-0.070	
55	5	50	56	55	4	51	56	307672.590	0.026	0.020
55	5	50	54	55	4	51	54	307672.590	0.014	0.020
56	3	53	57	56	2	54	57	276839.630	-0.007	0.005
56	3	53	55	56	2	54	55	276839.630	0.017	0.005
56	3	53	56	56	2	54	56	276841.036	0.034	
56	5	51	56	56	4	52	56	302587.279	-0.047	
56	5	51	57	56	4	52	57	302587.995	0.036	0.031
56	5	51	55	56	4	52	55	302587.995	0.025	0.031
57	4	53	56	56	5	52	55	259600.666	0.044	0.035
57	4	53	58	56	5	52	57	259600.666	0.026	0.035
57	4	53	57	56	5	52	56	259601.702	0.019	
57	3	54	58	57	2	55	58	287965.501	0.002	0.014
57	3	54	56	57	2	55	56	287965.501	0.026	0.014
57	3	54	57	57	2	55	57	287966.847	-0.028	
57	5	52	57	57	4	53	57	297582.603	-0.065	
57	5	52	58	57	4	53	58	297583.243	-0.033	-0.038
57	5	52	56	57	4	53	56	297583.243	-0.044	-0.038
58	5	53	59	58	4	54	59	292717.471	-0.012	-0.017

Continued on next page

Table S6 – *Continued from previous page*

J'	K_a'	K_c'	F'	J	K_a	K_c	F	Frequency	Obs.-Calc.	Average
58	5	53	57	58	4	54	57	292717.471	-0.022	-0.017
59	5	54	60	59	4	55	60	288050.808	0.057	0.052
59	5	54	58	59	4	55	58	288050.808	0.047	0.052
59	4	55	58	58	5	54	57	295798.157	-0.020	-0.028
59	4	55	60	58	5	54	59	295798.157	-0.037	-0.028
59	4	55	59	58	5	54	58	295799.266	0.003	
60	4	56	61	60	3	57	61	234094.508	0.027	0.032
60	4	56	59	60	3	57	59	234094.508	0.037	0.032
60	5	55	61	60	4	56	61	283643.453	-0.015	-0.019
60	5	55	59	60	4	56	59	283643.453	-0.023	-0.019
61	5	56	62	61	4	57	62	279555.197	-0.072	-0.076
61	5	56	60	61	4	57	60	279555.197	-0.079	-0.076
62	4	58	63	62	3	59	63	246006.411	0.008	0.014
62	4	58	61	62	3	59	61	246006.411	0.020	0.014
63	4	59	64	63	3	60	64	253005.303	-0.106	-0.099
63	4	59	62	63	3	60	62	253005.303	-0.093	-0.099
63	4	59	63	63	3	60	63	253006.278	0.056	
64	4	60	65	64	3	61	65	260678.091	-0.034	-0.028
64	4	60	63	64	3	61	63	260678.091	-0.021	-0.028
64	4	60	64	64	3	61	64	260679.075	0.076	
65	4	61	66	65	3	62	66	269001.011	-0.033	-0.026
65	4	61	64	65	3	62	64	269001.011	-0.019	-0.026
65	4	61	65	65	3	62	65	269002.057	0.084	
66	4	62	67	66	3	63	67	277944.858	-0.004	0.004
66	4	62	65	66	3	63	65	277944.858	0.011	0.004
66	4	62	66	66	3	63	66	277945.767	-0.073	
67	4	63	68	67	3	64	68	287474.647	-0.037	-0.030
67	4	63	66	67	3	64	66	287474.647	-0.022	-0.030
67	4	63	67	67	3	64	67	287475.736	0.027	
75	5	70	76	75	4	71	76	281948.484	-0.028	-0.025
75	5	70	74	75	4	71	74	281948.484	-0.022	-0.025
76	5	71	77	76	4	72	77	287530.460	-0.023	-0.019
76	5	71	75	76	4	72	75	287530.460	-0.015	-0.019
61	4	57	62	61	3	58	62	239698.828	0.047	0.053
61	4	57	60	61	3	58	60	239698.828	0.058	0.053
42	3	40	43	42	2	41	43	299106.220	-0.006	0.006
42	3	40	41	42	2	41	41	299106.220	0.018	0.006

Continued on next page

Table S6 – *Continued from previous page*

J'	K'_a	K'_c	F'	J	K_a	K_c	F	Frequency	Obs.-Calc.	Average
42	3	40	42	42	2	41	42	299107.301	0.068	
58	3	55	59	58	2	56	59	299352.318	-0.012	-0.001
58	3	55	57	58	2	56	57	299352.318	0.011	-0.001
58	3	55	58	58	2	56	58	299353.695	-0.017	
40	1	39	41	40	0	40	41	299553.741	0.014	0.036
40	1	39	39	40	0	40	39	299553.741	0.057	0.036
40	1	39	40	40	0	40	40	299555.468	0.030	
15	2	13	15	14	1	14	14	299804.715	-0.060	
29	2	28	29	28	1	27	28	318032.097	0.043	
68	4	64	69	68	3	65	69	297550.361	-0.044	-0.036
68	4	64	67	68	3	65	67	297550.361	-0.028	-0.036
68	4	64	68	68	3	65	68	297551.523	0.054	
30	2	29	31	29	1	28	30	323190.287	-0.005	-0.022
30	2	29	29	29	1	28	28	323190.287	-0.038	-0.022
